# Generic outcome set for the international registry on Laser trEAtments in Dermatology (LEAD): a protocol for a Delphi study to achieve consensus on *what* to measure

**DOI:** 10.1136/bmjopen-2020-038145

**Published:** 2020-06-28

**Authors:** Frederike Fransen, Phyllis Spuls, Murad Alam, Ashraf Badawi, Pablo Boixeda, Merete Haedersdal, Iltefat Hamzavi, Lene Hedelund, Kristen M Kelly, Tara Kono, Hans Joachim Laubach, Woraphong Manuskiatti, Leonardo Marini, Keyvan Nouri, Uwe Paasch, Thierry Passeron, Cecilia A.C. (Sanna) Prinsen, Ines Verner, Albert Wolkerstorfer

**Affiliations:** 1 Dermatology, Amsterdam UMC, Amsterdam, Noord-Holland, The Netherlands; 2 Department of Dermatology, Amsterdam Public Health, Infection and Immunity, Amsterdam University Medical Center, Amsterdam, The Netherlands; 3 Department of Dermatology, Feinberg School of Medicine, Northwestern University, Chicago, Illinois (IL), United States; 4 Department of Dermatology, Northwestern Memorial Hospital, Arkes Family Pavilion, Chicago, Illinois (IL), United States; 5 Dermatology Unit, Department of Medical Applications of Lasers, National Institute of Laser Enhanced Sciences, Cairo University, Giza, Egypt; 6 Dermatology Department, Ramón y Cajal Hospital, Madrid, Spain; 7 Dermatology, Copenhagen University Hospital Bispebjerg, Copenhagen, Denmark; 8 Massachusetts General Hospital, Harvard Medical School Boston, Boston, United States; 9 Department of Dermatology, Henry Ford Hospital, Detroit, Michigan, USA; 10 Dermatology, Aarhus Universitetshospital, Aarhus, Denmark; 11 Beckman Laser Institute, University of California, Irvine, California, USA; 12 Department of Plastic and Reconstructive Surgery, Tokai University School of Medicine, Isehera, Japan; 13 Dermatology and Venereology, Hopitaux Universitaires de Geneve, Geneva, Switzerland; 14 Faculty of Medicine Siriraj Hospital, Department of Dermatology, Mahidol University, Bangkok, Thailand; 15 Dermatology, SDC—The Skin Doctors’ Center, Trieste, Italy; 16 Dermatology and Cutaneous Surgery, University of Miami School of Medicine, Miami, Florida, USA; 17 University of Leipzig, Leipzig, UK; 18 Dermatology, Centre Hospitalier Universitaire de Nice, Nice, Provence-Alpes-Côte d'Azu, France; 19 Department of Epidemiology and Biostatistics, Amsterdam Public Health research institute, Amsterdam UMC, VU University Medical Center, Amsterdam, The Netherlands; 20 Dermatology, Verner clinic, Tel Aviv, Israel

**Keywords:** laser therapy, dermatology, surgical dermatology

## Abstract

**Introduction:**

While laser technology has expanded the armamentarium of treatment for various skin diseases during the past years, heterogeneity in study outcomes hampers comparability and appropriate evidence synthesis. Part of these issues can be addressed by developing a generic outcome set. Using the Delphi method, this study aims to seek consensus between key stakeholders on relevant generic outcomes (*what* to measure) for implementation in the international registry on Laser trEAtments in Dermatology (LEAD). The registry is focused on collecting research data on various laser treatments for skin disorders.

**Methods and analysis:**

By reviewing the literature and involvement of key stakeholder groups and adult patients in need or after laser surgery and health professionals, a preliminary list of outcomes will be generated and categorised into domains. Using these outcomes, an international three-round Delphi study will be performed to rate the importance of outcomes in the selection of a generic outcome set. Participants are allowed to provide new outcomes to the preliminary list for revisions during the first Delphi round. Finally, results will be discussed during a consensus meeting to agree on generic outcomes to be used in the LEAD registry.

**Ethics and dissemination:**

An ethics approval was not applicable (W19_290 # 18.336). The study is registered with the Cochrane Skin Core OUtcome Set INitiative) and the Core Outcome Measures in Effectiveness Trials initiative. Procedures will be conducted according to the Declaration of Helsinki. The findings will be disseminated through peer-reviewed publications and conference presentations.

**Strengths and limitations of this study**

- This protocol outlines the first international consensus effort to develop a generic outcome set for use in the international Laser trEAtments in Dermatology registry.
- With advances in laser technology, considering outcomes of importance (*what* to measure) to patients and health professionals is crucial.
- A comprehensive systematic review will explore which outcomes are used and reported in existing studies on laser treatments.
- The Delphi procedure requires three survey rounds and involves a large group of stakeholders across various disciplines and geographical areas including patients, reflecting different viewpoints.

## Introduction

During the past decades, modifications in laser technology have further widened its scope and greatly expanded the cutaneous laser surgeon’s armamentarium.^1 2^ Today, there are many medical indications in dermatology, encompassing vascular, pigmented, inflammatory, metabolic or infectious lesions, benign tumours, scars and hair follicle-related skin conditions that are regularly—and sometimes exclusively—treated with lasers.^1–3^ Many of these disorders meet the criteria of an orphan disease.

The diversity in laser devices and the spectrum of medical indications pose unique research challenges for clinical decision-making in laser therapy. Because most laser physicians are not exposed to large numbers of patients receiving laser treatments for uncommon indications, knowledge on the most effective laser treatment, including safety and used regimen, is unclear. The current evidence for most of these specific skin conditions is sporadic at best, consisting mostly of case reports and case series and only a very small number of randomised controlled trials (RCTs).^4 5^ Moreover, most often only isolated successes are reported while cases that failed to respond are not published, leading to publication bias.^6^

Another issue hampering evidence synthesis is heterogeneity of outcome definition, measurement and reporting in laser research. Patient-reported outcomes, such as ‘patient experience of laser treatments’ and ‘health-related quality of life’, are often not reported and together with selective outcome reporting in laser research, it is all a serious threat to comparative effectiveness research as it limits the ability to compare, contrast and combine individual studies.^7 8^ As a result, this hampers to draw meaningful conclusions and guidance to inform clinical decision-making.^9 10^

To overcome this issue in the field of laser dermatology, the development of the International Laser Treatment (Laser trEAtments in Dermatology (LEAD)) Registry has been proposed to initiate collaborative data pooling of a wide range of skin disorders. The development of a registry may be the key to the lack of solid evidence for LEAD; however, well-defined standardised and generic outcomes are required for its establishment.

To address the variations in outcome reporting, organisations such as the Core Outcome Measures in Effectiveness Trials Initiative (COMET) bring together researchers interested in developing a standardised set of core outcomes in various health-related fields.^11^ A core outcome set (COS) is defined as an agreed minimum set of outcomes that should be measured and reported in all clinical trials for a specific health condition, including methods used to measure these core outcomes.^10 12^ Throughout this report, the definition of ‘outcome’ refers to a single construct that can be measured as a stand-alone item (eg, ‘erythema’), while the term ‘outcome domain’ or ‘domain’ is an umbrella term for a group of associated outcomes (e.g. ‘signs as assessed by physician’). Furthermore, the outcome instrument refers to how the outcomes are measured. Although a COS is recommended for clinical trials, they can also be developed for routine clinical practice, and for registries.^10 12^ In 2015, the international, multidisciplinary working group, the Cochrane Skin Group—Core OUtcome Set INitiative (CS-COUSIN) has been established.^13^ The organisation supports dermatology-specific initiatives to develop and implement a COS by building on experiences of the Harmonising Outcome Measures for Eczema initiative, which developed a roadmap to guide the process of COS development and implementation.^14^ Currently, 17 COS initiatives have been supported by CS-COUSIN in dermatology. These projects involve 26 different skin diseases, such as acne, atopic eczema, hidradenitis suppurativa, melanoma, nail psoriasis, rosacea and vitiligo.^11 15^ However, with hundreds of different and mostly unrelated dermatoses that are treated with lasers in the field of laser dermatology, the need for a generic outcome set (GOS) is commanding. Therefore, we focus on developing a GOS (*what* to measure) for the purpose of the LEAD registry. The GOS is intended to be applied for the assessment of various, unrelated skin diseases that are treated with different types of lasers.

In summary, there is an urgency of using the same generic outcomes in laser therapy. Hence, establishing consensus on the relevant outcomes for the LEAD registry will promote clinical researchers to use outcomes chosen by consensus that are relevant to patients and clinicians. The use of generic outcomes support data synthesis for many diseases in dermatology. The protocol outlines the context, scope and methods for the development of a GOS to be implemented in the LEAD registry.

## Aims and objectives

### Aim

The aim of this study is to reach consensus between various stakeholders on generic outcomes relevant for the LEAD registry.

### Objectives

Our study objectives are:

1. To identify outcomes that have previously been used and reported in RCTs, cohort studies, case-control studies and case series from a literature review and classify these outcomes into domains according to the COMET taxonomy.
2. To reach consensus between stakeholders on the outcomes of a GOS to be implemented in the LEAD registry.

### Scope and applicability of outcomes

The registry is envisioned to suit all types of laser interventions for skin disorders in dermatology including vascular, pigmented or inflammatory lesions, benign tumours, scars and hair follicle-related skin conditions treated with lasers. The GOS is intended for use in the LEAD registry, with the focus on prospectively recording the effectiveness and safety of cutaneous non-cosmetic laser interventions. Therefore, we excluded laser-assisted drug delivery, low laser level therapy, body contouring, skin tightening, hair removal, rejuvenation and antiageing procedures. Furthermore, because of the distinctive mode of action and use in daily clinical practice, laser-assisted drug delivery, low laser level therapy and laser procedures for (leg) veins were excluded.

## Methods and analysis

### Research group

The steering committee (FF, PS, AW, MA, AB, PB, IH, MH, LH, KMK, TK, HJL, WM, LM, KN, UP, TP, CP, IV) provide input at critical points of the study such as protocol development, stakeholder recruitment, consensus process and the consensus meeting. Three members of the steering committee (FF, PS, AW) coordinate the overall project, ensure methodological quality of the project and make key decisions. All members of the steering committee will participate in the Delphi procedure as well as in the final consensus meeting. The steering committee has representatives from The Netherlands, Denmark, Egypt, France, Germany, Israel, Italy, Japan, Spain, Switzerland, Thailand and USA, with extensive expertise in various laser treatments, outcomes research and clinical research. A list of all members of the steering committee is given in online supplementary file 1.

10.1136/bmjopen-2020-038145.supp1 Supplementary data

### Study design

**Figure 1:**
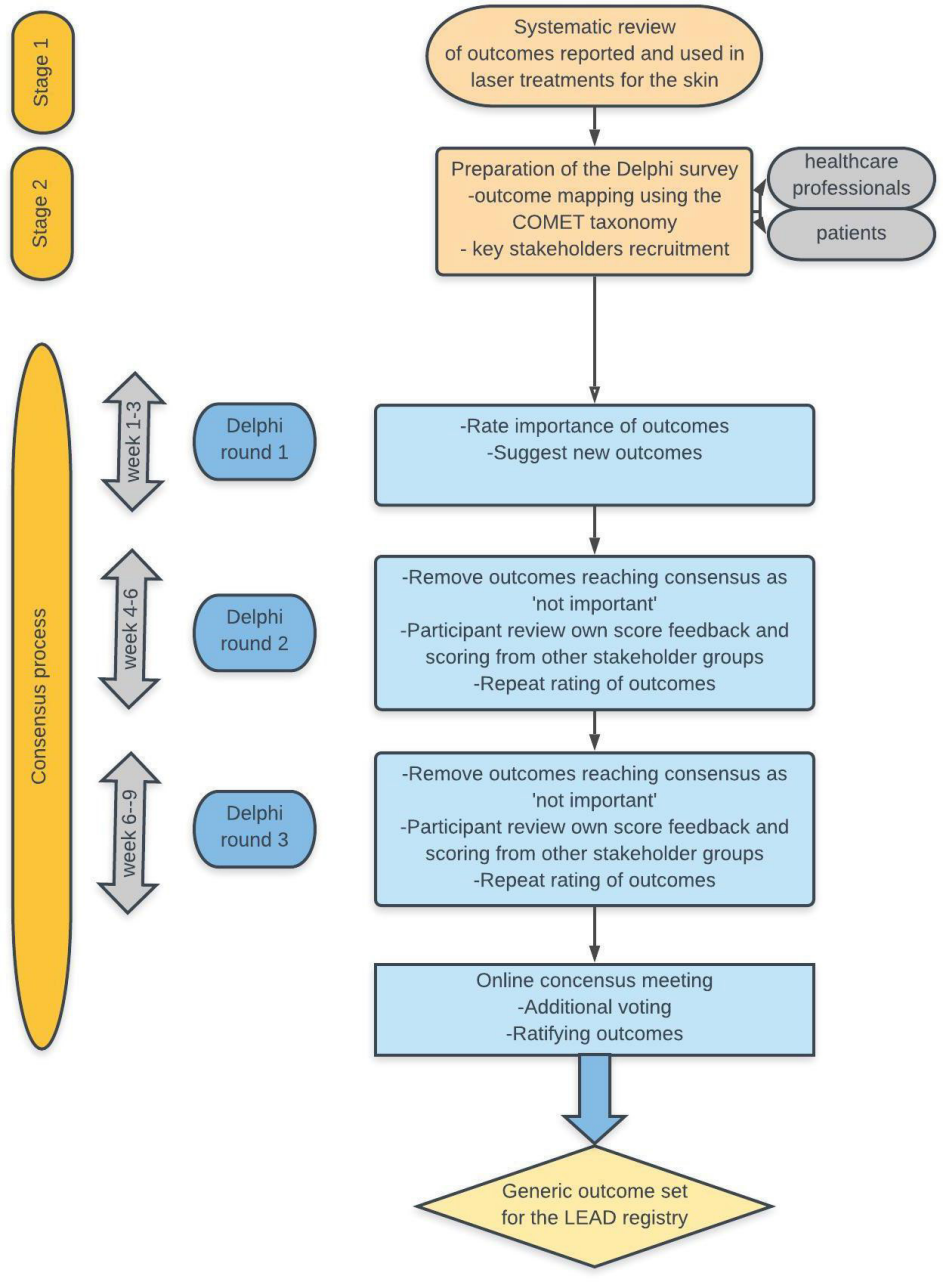
Flow diagram outlining the development of a generic outcome set for the Laser trEAtments in Dermatology (LEAD) registry. Preparatory stages and process of consensus for relevant generic outcomes are summarised. COMET, Core Outcome Measures in Effectiveness Trials Initiative.

Figure 1 provides a brief overview of the stepwise approach with different research methods. The study consists of the following two phases:

Phase 1: identification of potential outcomes important in laser treatments by means of a

1. A systematic review to form the preliminary list of outcomes for the Delphi survey.
2. Classification of outcomes into domains according to the COMET taxonomy.

Phase 2: A consensus process involving key stakeholders who are able to suggest additional outcomes during the first round and who will rate the importance of outcome for reaching consensus on the GOS by means of a

1. Three-round Delphi survey.
2. Expert consensus meeting. Attended by representatives of all stakeholder groups.

This study is registered with the CS-COUSIN and COMET initiative.^11 16^ Results of the consensus study will be reported according to the Core Outcome Set-STAndards for Reporting.^17^

### Phase 1: identification of potential outcomes and domains

#### Phase 1.1: systematic literature review

The first phase of the study is to identify which outcomes should be measured and reported in a registry on laser treatments for skin disorders (what to measure: the GOS, see definitions in online supplementary file 2). An SR will be performed to explore existing outcomes that are used in laser studies. According to the COMET guidelines,^18^ searches will be performed in the following database: Medline and Embase. Articles between January 2013 and December 2017 will be retrieved. The electronic search strategy is detailed in online supplementary file 3. A recent 5-year time period has been selected for the search so that outcomes extracted represent the practice of present-day laser research. The inclusion and exclusion criteria are presented in table 1. Two reviewers will select articles and extract the data independently. Disagreement will be resolved by discussion and by consulting a third review author if necessary. The following data will be extracted from the selected articles in data extraction tables : authors, years of publication, country, cutaneous indications for treatment and type of laser treatments. We will assess what outcomes and outcome measurement instrument are used, consistency in outcomes, number of times an outcome was used, consistency in classification used.

10.1136/bmjopen-2020-038145.supp2 Supplementary data

10.1136/bmjopen-2020-038145.supp3 Supplementary data

**Table 1:** Inclusion and exclusion criteria for literature review

	Inclusion criteria	Exclusion criteria
Patient population and indication	Studies including patients age 18 and older with vascular, pigmented, inflammatory, metabolic or infectious lesions, benign tumours and hair follicle-related skin conditions treated with lasers	Non-humans flebological skin conditions Laser-assisted drug delivery, low laser level therapy, body contouring, skin tightening, hair removal, rejuvenation and antiageing
Study design	RCTs, cohort studies, case-control studies, case series	In vitro studies, systematic reviews, abstracts and expert opinions, case reports
Intervention	Any type of laser treatment for vascular, pigmented or inflammatory lesions, benign tumours and hair follicle-related skin conditions.	Laser-assisted drug delivery, low laser level therapy, laser therapy for leg veins and cosmetic interventions (see scope of outcomes)
Outcomes		Non-clinical outcomes For example, biochemical outcomes, imaging, confocal laser, histology
Publication	All studies are conducted between 2013 and 2017	

RCT, randomised controlled trial.

#### Phase 1.2: classification of outcomes into domains

Subsequently, data will be classified according to the standardised taxonomy for outcomes proposed by the COMET initiative.^19^ This taxonomy encompasses 38 domains within 5 core areas: mortality/survival; physiological/clinical; life impact; resource use; adverse events.

Outcomes and their classification in domains will be discussed with three members (FF, PS, AW) of the steering committee. The preliminary list of outcomes classified to domains will be included in the consensus process.

### Phase 2: consensus process

#### Phase 2.1: Delphi procedure

For investigating crucial outcomes in context of the LEAD registry, a Delphi study will be conducted. The Delphi is based on a structured process for gathering and condensing knowledge from key stakeholder groups by means of three rounds with a series of questionnaires 20 The procedure will consist of three online rounds (figure 1).

### Participants

The involvement of a variety of stakeholders is a key part for the identification of outcomes and strongly recommended by methodologists.^21^

The following representatives from four international key stakeholder groups are involved in the process of reaching consensus on outcomes:

1. Patients of age 18 with vascular, pigmented, inflammatory, metabolic or infectious lesions, benign tumours and hair follicle-related skin conditions treated by lasers.
2. Patient representatives involved in patient associations that raise awareness on the impact of vascular, pigmented, inflammatory, metabolic or infectious lesions, benign tumours and hair follicle-related skin conditions.
3. Healthcare professionals: laser experts who treat patients with vascular, pigmented or inflammatory, metabolic or infectious lesions, benign tumours, hair follicle-related skin conditions and who are involved in research on laser treatments.
4. Healthcare professionals: general physicians who treat patients with dermatological indications.

### Panel size and recruitment

There is no robust guidance for calculating the number of participants needed for a Delphi study and expectations are based on COMET Initiative guidelines and previous literature.^16 22 23^ As there are various stakeholder groups involved in the Delphi procedure, we will recruit as many international representatives as possible from each group. All potential participants will be invited with a letter explaining the aims and details of the study and the rationale and importance of completing the entire Delphi process. Respondents who agree to take part will be assigned a unique identification number. Furthermore, each member of the steering committee will be asked to cascade the link of the survey to three other physicians in their network. Patients and patient representatives will be recruited from national and international support groups for skin diseases treated with lasers and can be found in online supplementary file 4. In addition, laser experts from the steering committee will be asked to recruit three patients with different skin conditions treated with lasers in their centre. To make sure that we involve skin diseases of different categories, laser experts will indicate the diagnosis of the patients that are recruited. By sending the survey invitation to experts and patient support groups from different continents, we aim to reflect a broad range of patients and health professionals with diverse backgrounds and experiences. For each round, the number of participants invited and those who completed the surveys will be documented. The participants will have 3 weeks to complete each round. We will send personal reminder emails to those who did not respond after 7 and 14 days to increase the response rate.

10.1136/bmjopen-2020-038145.supp4 Supplementary data

### Delphi survey

Participants will be divided into a group of patient and a group of health professional, leading to separate scoring of outcomes. All participants will be asked to rate the importance of each of the outcomes using the Grading of Recommendations Assessment, Development and Evaluations approach. The scale will range from 1 to 9 and will be categorised as follows: 1–3 ‘not important’; 4–6 ‘important but not critical’; and 7–9 ‘critical’.^24 25^ If participants feel unable to rate or provide feedback, they can select ‘unable to score’.

### Delphi rounds

#### Delphi round 1

During the first round of the Delphi survey, baseline characteristics (age, gender, country of practice) will be obtained from all participants. Patients will be asked for their medical indication and type of laser treatment, and whether any complications have occurred during treatment. Health professionals will be asked their specialty (laser dermatology, general dermatology or other), workplace (academic, teaching hospital or non-teaching hospital) and years in practice. Next, participants will be asked to score listed outcomes and will have the option to suggest any additional outcomes that are not yet presented in the preliminary list.

#### Delphi rounds 2 and 3

In the second and third Delphi rounds, all participants will receive feedback on the scores of the previous round in both the patient and the health professional group. The outcomes from the previous rounds will be presented with the median scores from each stakeholder group combined with a histogram showing the scoring distribution. Subsequently, participants will be asked to score all outcomes for which consensus has not been reached, in the same manner as in the first Delphi round. Outcomes for which there was only consensus within a single stakeholder group will also be shown to the other stakeholder group to evaluate whether consensus can be achieved in both stakeholder groups.

### Definition of consensus

The definition of consensus is presented in table 2. ‘Consensus in’ is defined as approval of the outcome by the vast majority (70 %) of all stakeholder groups that score 7, 8 or 9 with fewer than the minority (15 %) of panellists scoring 1–3. On the contrary, ‘consensus out’ is defined as 70% or more of all stakeholder groups scoring as 1 to 3 and less than 15% scoring as 7 to 9.^12^ After three e-Delphi rounds, outcomes will be classified as ‘consensus in’ (consensus on the importance of the outcome), ‘consensus out’ (no consensus on the importance, or consensus on non-importance) or ‘no consensus’ (consensus on the importance in only one or or no consensus).

**Table 2:** Definitions of consensus for identifying generic outcomes for the Laser trEAtments in Dermatology registry

Consensus category	Clarification	Definition
Consensus in	Outcome should be included in the registry	70% of stakeholder groups scoring as 7–9 and <15% of stakeholder groups scoring as 1–3
Consensus out	Outcome should not be included in the registry	70% or more of stakeholder groups scoring as 1–3 and <15% of stakeholder groups scoring as 7–9
No consensus	Hesitation about relevance of outcome to be included in the registry	Anything other

#### Phase 2.2: determination of the GOS during the expert consensus meeting

In case complete consensus is reached in the Delphi procedure on the outcomes of the GOS, no formal consensus meeting will be organised. However, the results of the Delphi will be discussed with three members of the steering committee (FF, PS, AW) to check misconceptions in the Delphi method and to safeguard a well-defined GOS. For outcomes for which consensus definition during the Delphi has not been reached, we invite 15 participants from across all stakeholder groups to participate in an online expert consensus meeting within 2 months after the close of round 3. The primary goal of the meeting is discussing the ‘no consensus’ outcomes. Consensus results from the Delphi can be reversed in this meeting if reasons are very strong and clear.

### Patient and public involvement

Patient and public were not involved in the development of this study protocol. However, patients will be involved and included within the Delphi procedure as expert group. Consensus methodology will ensure that the opinions and preferences of patients will be given the same weighting as those of the laser experts and health professionals. Furthermore, patients will participate in the final consensus meeting. We disseminate the main results to study participants and patients by email which will include a copy of the final outcomes of the GOS. In addition, where approval has been given, participants (including members of the public) will be named as contributors in the acknowledgements section.

## Discussion

By the end of this study, we hope to reach consensus on a GOS that could be implemented in an international registry with a research focus, that collects data of rare skin diseases treated by lasers. Analysis of registry data provides insight into effectiveness and safety of different laser treatments across many skin diseases, laser centres and countries.

There are several strengths using the Delphi method for this study. First, the Delphi method allows to recruit a large number of laser experts, physicians and patients from diverse regions globally. The diversity in the experts’ backgrounds and expertise ensures maximum impact of the results. Second, the Delphi method is the accurate tool in consensus processes in various stakeholder groups as individuals are able to express their own opinions and feedback can be provided in a controlled anonymous way. This means that there is room for individual disagreement but also consideration of the answers given by other individuals and stakeholder groups as a whole. However, there are also limitations of the Delphi method. Results are dependent on the composition of the participants. There is a risk of relative uneven representations among patients, but also health professionals. Especially, when focusing on a specific group of rare skin diseases, selection bias could result in insufficient representation of other skin disorders. We request health professionals of the steering committee to recruit patients with three different skin disorders. Through this method, we hope to ensure that all subgroups including vascular, pigmented, metabolic, inflammatory lesions, benign tumours and hair follicle-related skin conditions will be adequately involved. For patients, it might be a barrier to imagine what is important to be included in a registry for a broad range of diseases, rather than one disease that is important to themselves. We will stress the importance of agreeing on a GOS for all diseases in each round of the Delphi survey and consensus meetings. Photographs will be included to illustrate the variety of skin disorders that are involved. To provide the highest possible input, we will extend our invitation to take part in the Delphi survey to patients and health professionals in Africa, Asia, South-America, Australia, in addition to Europe and North-America. With support from all panel members, we hope to ensure that the LEAD registry will be internationally relevant, accepted and ready to use.

### Trial status

The identification of generic outcomes for registry use is ongoing and in the initial phase. A systematic review has been performed to explore current outcomes used and reported in laser dermatology. We are currently preparing to recruit participants for the Delphi study. The generic outcomes are expected to be implemented in the laser registry in 2020.

## Ethics and dissemination

The medical research ethics committee of the Academic Medical Center Amsterdam confirmed that the Dutch Medical Research Involving Human Subjects Act does not apply to this study (W19_290 # 18.336) and that complete approval of this study by the committee is not necessary. All participants involved in the Delphi study will be asked for their consent before taking part. All procedures will be conducted according to the Declaration of Helsinki. All results from the consensus study will be reported in peer-reviewed indexed journals. The data will be presented at conferences chosen to reach a wide range of knowledge users.

## Supplementary Material

Reviewer comments

Author's manuscript
