# Supplementary material for: Generic outcome set for the international registry on Laser trEAtments in Dermatology (LEAD): a protocol for a Delphi study to achieve consensus on what to measure
Source: BMJ Open. 2020 Jun 28;10(6):e038145. doi: 10.1136/bmjopen-2020-038145 (PMC7322331; doi:10.1136/bmjopen-2020-038145)
Supplement: Supplementary data [file bmjopen-2020-038145supp001.pdf]

**SUPPLEMENTARY FILE 1**

**LEAD Registry : Steering Committee**

<u>*Coordination team*</u>

Frederike Fransen (the Netherlands)

Albert Wolkerstorfer (the Netherlands)

Phyllis Spuls (the Netherlands)

In addition to the coordinaton team , the LEAD registry Steering Committee includes:

Murad Alam (US),Ashraf Badawi (Egypt), Pablo Boixeda (Spain), Iltefat Hamzavi (US), Merete Haedersdal (Denmark), Lene Hedelund (Denmark), Kristen Kelly (US), Taro Kono (Japan), Hans-Joachim Laubach (Switzerland), Woraphong Manuskiatti (Thailand), Leonardo Marini (Italy), Keyvan Nouri (US), Uwe Paasch (Germany), Thierry Passeron (France), Sanna Prinsen (The Netherlands), Ines Verner (Israel)

**Author information list**

Frederike Fransen (the Netherlands)

*Amsterdam UMC, Department of Dermatology, 9, 1105 AZ, Amsterdam, the Netherlands.*

***f.fransen@amsterdamumc.nl***

Albert Wolkerstorfer (the Netherlands)

*Amsterdam UMC, Department of Dermatology, 9, 1105 AZ, Amsterdam, the Netherlands*

***a.wolkerstorfer@amsterdamumc.nl***

Phyllis Spuls (the Netherlands)

*Amsterdam UMC, Department of Dermatology, 9, 1105 AZ, Amsterdam, the Netherlands*

***ph.i.spuls@amsterdamumc.nl***

Murad Alam (US)

*Department of Dermatology, Feinberg School of Medicine, Northwestern University, Chicago, IL, USA. m-alam@northwestern.edu.*

*Department of Dermatology, Northwestern Memorial Hospital, Arkes Family Pavilion, 676 N Saint Clair Suite 1600, Chicago, IL, 60611, USA. m-alam@northwestern.edu*

**m-alam@northwestern.edu**

Ashraf Badawi (Egypt)

*Dermatology Unit, Department of Medical Applications of Lasers (MAL), National Institute of Laser Enhanced Sciences, Cairo University, Giza, Egypt*

**ashrafbadawi@hotmail.com**

Pablo Boixeda (Spain)

*Dermatology Department, Ramón y Cajal Hospital, Madrid, Spain*

**pboixeda@gmail.com**

Iltefat Hamzavi (US)

*Department of Dermatology, Henry Ford Hospital, Detroit, MI, USA*

**IHamzavi@hamzavi.com**

Merete Haedersdal (Denmark)

*Massachusetts General Hospital, Harvard Medical School Boston, USA*

*University of Copenhagen, Bispebjerg Hospital, Denmark*

**mhaedersdal@dadlnet.dk**

Lene Hedelund (Denmark)

*Department of Dermatology, Aarhus University Hospital, Denmark*

**lenehede@rm.dk**

Kristen Kelly (US)

*Beckman Laser Institute, University of California, Irvine, California, USA*

**kmkelly@uci.edu**

Taro Kono (Japan)

*Department of Plastic and Reconstructive Surgery, Tokai University School of Medicine, Isehara, Japan.*

**tkono@tokai-u.jp**

Hans-Joachim Laubach (Switzerland)

*Department of Dermatology and Venereology, Geneva University Hospitals (HUG), Switzerland.*

**hlaubach@esld.eu**

Woraphong Manuskiatti (Thailand)

*Faculty of Medicine Siriraj Hospital, Department of Dermatology , Mahidol University , Bangkok , Thailand*

**woraphong.man@mahidol.ac.th**

Leonardo Marini (Italy)

*SDC - The Skin Doctors' Center, Trieste, Italy*

**leonardo.marini@skindoctors.it**

Keyvan Nouri (US)

*Dermatology and Cutaneous Surgery, University of Miami School of Medicine, 1475 NW 12th Ave., Miami, FL, 33136, USA*

**KNouri@med.miami.edu**

Uwe Paasch (Germany)

*Department of Dermatology, Venereology and Allergy, University of Leipzig*

**uwe.paasch@hautclinicum.de**

Thierry Passeron (France)

*University of Côte d'Azur, University Hospital Nice, Department of Dermatology, Nice, France*

*University of Côte d'Azur, Centre Méditéranéen de Médecine Moléculaire (C3M), INSERM U1065, team 12, Nice, France*

**Thierry.Passeron@unice.fr**

Sanna Prinsen (The Netherlands)

*Department of Epidemiology and Biostatistics, Amsterdam Public Health research institute, Amsterdam UMC, Vrije Universiteit Amsterdam, The Netherlands.*

**c.prinsen@vumc.nl**

Ines Verner (Israel)

*Verner Clinic, Tel Aviv, Israel.*

***ines.verner@gmail.com***
