# Supplementary material for: Generic outcome set for the international registry on Laser trEAtments in Dermatology (LEAD): a protocol for a Delphi study to achieve consensus on what to measure
Source: BMJ Open. 2020 Jun 28;10(6):e038145. doi: 10.1136/bmjopen-2020-038145 (PMC7322331; doi:10.1136/bmjopen-2020-038145)
Supplement: Supplementary data [file bmjopen-2020-038145supp002.pdf]

SUPPLEMENTARY FILE 2

The definitions for COS, outcome, outcome instruments and outcome parameters according to Prinsen *et al*. (2014). [1]

Definitions
Similar constructs are defined differently across several research groups such as COMET, OMERACT, and HOME. As there is currently no consensus on the definitions, we would like to explicitly state the definitions that are being used in the COMET Delphi study in order to avoid any possible misinterpretations.

Core outcome set (COS)
A COS is an agreed minimum set of outcomes that should be measured and reported in all clinical trials of a specific disease or trial population. A COS includes all relevant outcomes of a specific health condition within a specified setting (the OMERACT definition refers to 'core domain set' whereas the HOME definition refers to 'core outcome domains').

Generic core outcome set (GOS)
A GOS is an agreed minimum set of *generic* outcomes that should be measured and reported in all clinical trials of a specific disease or trial population. In this study, the GOS is intended to be applied for the assessment of various, unrelated skin diseases that are treated with different types of lasers.

Outcome and outcome domain.
Throughout this report, the definition of "outcome" refers to a single construct that can be measured as a standalone item (e.g. 'erythema'), while the term "outcome domain" or "domain" is an umbrella term for a group of associated outcomes (e.g. 'signs as assessed by physician').

Outcome measurement instrument
An outcome measurement instrument refers to how the outcome is being measured (the tool used to assess the outcome). An outcome measurement instrument can be a single question, a questionnaire, a performance-based test, a physical examination, a laboratory measurement, an imaging technique, and so forth (the HOME definition refers to 'outcome measure').

Reference

1 Prinsen CAC, Vohra S, Rose MR, *et al.* Core Outcome Measures in Effectiveness Trials (COMET) initiative: Protocol for an international Delphi study to achieve consensus on how to select outcome measurement instruments for outcomes included in a 'core outcome set'. *Trials* 2014;**15**. doi:10.1186/1745-6215-15-247
