# Supplementary material for: Generic outcome set for the international registry on Laser trEAtments in Dermatology (LEAD): a protocol for a Delphi study to achieve consensus on what to measure
Source: BMJ Open. 2020 Jun 28;10(6):e038145. doi: 10.1136/bmjopen-2020-038145 (PMC7322331; doi:10.1136/bmjopen-2020-038145)
Supplement: Supplementary data [file bmjopen-2020-038145supp003.pdf]

SUPPLEMENTARY FILE 3

Systematic review search strategies

Pubmed

1.“Skin” [Majr MeSH]

2.“cutaneous” [Majr MeSH]

3.“dermatology” [Majr MeSH]

4. “Skin Diseases”

5. 1 or 2 or 3 or 4

6.“laser” [Majr MeSH]

7.“alexandrite laser” [MeSH Terms]

8 “laser, pulsed dye” [MeSH Terms]

9. “er yag” [MeSH Terms]

10. “laser, nd yag” [MeSH Terms]

11. “laser, ruby” [MeSH Terms]

12. “laser, ysgg” [MeSH Terms]

13. “laser, argon” [MeSH Terms]

14. “laser, ktp” [MeSH Terms]

15. “laser, q switched” [MeSH Terms]

16. “laser, carbon dioxide” [MeSH Terms]

17. “laser, co2” [MeSH Terms]

18. “laser, diode” [MeSH Terms]

19. “thullium laser”

20. “fluoride laser”

21. “fractional laser”

22. “fractional CO2 laser”

23. “non-ablative fractional laser”

Fransen F, *et al. BMJ Open* 2020; 10:e038145. doi: 10.1136/bmjopen-2020-038145

24.“Humans[Mesh]

25. "last 5 years"[PDat]

26. 6 or 7 or 8 or 9 or 10 or 11 or 12 or 13 or 14 or 15 or 16 or 17 or 18 or 19 or 20 or 21 or 22 or 23

26. 5 and 26

Embase:

1. #1, Skin.mp. or exp skin/

2. #2, cutaneous.mp.

3. #3, dermatology.mp. or exp dermatology/

4. #4, skin diseases.mp. or exp skin disease/

5. #5, laser.mp. or exp laser/

6. #6, laser treatment.mp.

7. #7, laser therapy.mp.

8. #8, skin laser therapy.mp.

9. #9, exp argon laser/ or exp frequency doubled neodymium YAG laser/ or exp thulium YAG laser/ or exp dye laser/ or exp gallium aluminum arsenide laser/ or exp neodymium laser/ or exp pulsed dye laser/ or exp carbon dioxide laser/ or exp excimer laser/ or exp YAG laser/ or exp alexandrite laser/ or exp argon fluoride laser/ or exp gas laser/ or exp laser surgery/ or exp erbium YAG laser/

10. #10, nd YAG laser.mp.

11. #11, non-ablative fractional laser.mp.

12. #12, CO2 laser.mp.

13. #13, fractional CO2 laser.mp.

14. #14, carbon dioxide laser.mp. or exp carbon dioxide laser/

15. #15, q switched laser.mp.

16. #16, nd YAG laser.mp.

17. #17, exp symptom assessment/ or exp symptom/ or symptoms.mp.

18. #18, outcome assessment.mp. or exp outcome assessment/

19. #19, treatment outcome.mp. or exp treatment outcome/

20. #20, exp treatment outcome/ or exp outcome assessment/ or outcome.mp.

21. #1 or #2 or #3 or #4

22. #5 or #6 or #7 or #8 or #9 or #10 or #11 or #12 or #13 or #14 or #15 or #16

23. #17 or # 18 or #19 or #20

24. #21 and #22

25. #23 and #24

26. 25 and 2013:2017.(sa_year).

27. 26 and "human" [Subjects]
