# Supplementary material for: Generic outcome set for the international registry on Laser trEAtments in Dermatology (LEAD): a protocol for a Delphi study to achieve consensus on what to measure
Source: BMJ Open. 2020 Jun 28;10(6):e038145. doi: 10.1136/bmjopen-2020-038145 (PMC7322331; doi:10.1136/bmjopen-2020-038145)
Supplement: Supplementary data [file bmjopen-2020-038145supp004.pdf]

SUPPLEMENTARY FILE 4

A list of invited patient support groups for the Delphi survey

Name of Society
Hidradenitis Patiëntenvereniging (NL)
Nevus Netwerk Nederland (NL)
Nevus Outreach (US)
Nevus Support (AU)
Neurofibromatose Vereniging Nederland (NL)
The Neuro Foundation (UK)
Neurofibromatose Ireland Association (IE)
Vereniging Wijnvlek Sturgeweber syndroom (NL)
Schweizerischen Nuerofibromatose Vereinigung (CH)
Interessengemeinschaf Sturge-Weber-Syndrom (DE)
Sturge Weber Foundation Great Britain (UK)
Sturge-Weber-Foundation (US)
Vitiligo patientenvereniging (NL)
National Vitiligo Foundation (US)
