# Supplementary material for: Generic outcome set for the international registry on Laser trEAtments in Dermatology (LEAD): a protocol for a Delphi study to achieve consensus on what to measure
Source: BMJ Open. 2020 Jun 28;10(6):e038145. doi: 10.1136/bmjopen-2020-038145 (PMC7322331; doi:10.1136/bmjopen-2020-038145)
Supplement: Author's manuscript [file bmjopen-2020-038145.draft_revisions.pdf]

A generic outcome set for the international registry on Laser TrEAtments in Dermatology (LEAD): a protocol for a Delphi study to achieve consensus on what to measure

Journal:	*BMJ Open*
Manuscript ID	bmjopen-2020-038145
Article Type:	Protocol
Date Submitted by the Author:	29-Feb-2020

Complete List of Authors:	Fransen, Frederike; Amsterdam UMC , Dermatology<br>Spuls, Phyllis; Department of Dermatology, Amsterdam Public Health, Infection and Immunity, Amsterdam University Medical Center<br>Alam, Murad; Northwestern Medical Faculty Foundation,<br>Badawi, Ashraf; Dermatology Unit, Department of Medical Applications of Lasers (MAL), National Institute of Laser Enhanced Sciences, Cairo University<br>Boixeda, Pablo; Dermatology Department, Ramón y Cajal Hospital<br>Hamzavi, Iltefat; Department of Dermatology, Henry Ford Hospital, Detroit<br>Haedersdal, Merete; Copenhagen University Hospital Bispebjerg, Dermatology<br>Hedelund, Lene; Aarhus Universitetshospital, Dermatology<br>Kelly, Kristen ; Beckman Laser Institute, University of California<br>Kono, Tara; Department of Plastic and Reconstructive Surgery, Tokai University School of Medicine<br>Laubach, Hans Joachim; Hopitaux Universitaires de Geneve, Dermatology and Venereology<br>Manuskiatti, Woraphong; Faculty of Medicine Siriraj Hospital, Department of Dermatology , Mahidol University<br>Marini, Leonardo; SDC - The Skin Doctors' Center, Dermatology<br>Nouri , Keyvan ; Dermatology and Cutaneous Surgery, University of Miami School of Medicine, 1475 NW 12th Ave.,<br>Paasch, Uwe; University of Leipzig<br>Passeron, Thierry; Centre Hospitalier Universitaire de Nice, Dermatology<br>Prinsen, C; VU University Medical Center,, 19Department of Epidemiology and Biostatistics, Amsterdam Public Health research institute, Amsterdam UMC<br>Verner, Ines; Verner Clinic<br>Wolkerstorfer, Albert; Academic Medical Center (AMC), Dermatology

Keywords:	Laser therapy < DERMATOLOGY, DERMATOLOGY, Surgical dermatology < DERMATOLOGY

For peer review only - http://bmjopen.bmj.com/site/about/guidelines.xhtml

I, the Submitting Author has the right to grant and does grant on behalf of all authors of the Work (as defined in the below author licence), an exclusive licence and/or a non-exclusive licence for contributions from authors who are: i) UK Crown employees; ii) where BMJ has agreed a CC-BY licence shall apply, and/or iii) in accordance with the terms applicable for US Federal Government officers or employees acting as part of their official duties; on a worldwide, perpetual, irrevocable, royalty-free basis to BMJ Publishing Group Ltd ("BMJ") its licensees and where the relevant Journal is co-owned by BMJ to the co-owners of the Journal, to publish the Work in this journal and any other BMJ products and to exploit all rights, as set out in our licence.

The Submitting Author accepts and understands that any supply made under these terms is made by BMJ to the Submitting Author unless you are acting as an employee on behalf of your employer or a postgraduate student of an affiliated institution which is paying any applicable article publishing charge ("APC") for Open Access articles. Where the Submitting Author wishes to make the Work available on an Open Access basis (and intends to pay the relevant APC), the terms of reuse of such Open Access shall be governed by a Creative Commons licence – details of these licences and which Creative Commons licence will apply to this Work are set out in our licence referred to above.

Other than as permitted in any relevant BMJ Author's Self Archiving Policies, I confirm this Work has not been accepted for publication elsewhere, is not being considered for publication elsewhere and does not duplicate material already published. I confirm all authors consent to publication of this Work and authorise the granting of this licence.

For peer review only

A generic outcome set for the international registry on Laser TrEAtments in Dermatology (LEAD): a protocol for a Delphi study to achieve consensus on *what* to measure

Frederike Fransen[1], Phyllis I. Spuls[1], Murad Alam[2,3], Ashraf Badawi[4], Pablo Boixeda[5], Merete Haedersdal[6,7], Iltevat Hamzavi[8], Lene Hedelund[9], Kristen Kelly[10], Taro Kono[11], Hans-Joachim Laubach[12], Woraphong Manuskiatti[13], Leonardo Marini[14], Keyvan Nouri[15], Uwe Paasch[16], Thierry Passeron[17,18], Cecilia A.C. Prinsen[19], Ines Verner[20], Albert Wolkerstorfer[1]

[1] Department of Dermatology, Amsterdam Public Health, Infection and Immunity, Amsterdam University Medical Center, Amsterdam, the Netherlands

[2]Department of Dermatology, Feinberg School of Medicine, Northwestern University, Chicago, IL, USA. [3]Department of Dermatology, Northwestern Memorial Hospital, Arkes Family Pavilion, 676 N Saint Clair Suite 1600, Chicago, IL, 60611, USA. [4]Dermatology Unit, Department of Medical Applications of Lasers (MAL), National Institute of Laser Enhanced Sciences, Cairo University, Giza, Egypt. [5]Dermatology Department, Ramón y Cajal Hospital, Madrid, Spain. [6]Massachusetts General Hospital, Harvard Medical School Boston, USA. [7]University of Copenhagen, Bispebjerg Hospital, Denmark. [8]Department of Dermatology, Henry Ford Hospital, Detroit, MI, USA. [9]Department of Dermatology, Aarhus University Hospital, Denmark. [10]Beckman Laser Institute, University of California, Irvine, California, USA. [11]Department of Plastic and Reconstructive Surgery, Tokai University School of Medicine, Isehara, Japan. [12]Department of Dermatology and Venereology, Geneva University Hospitals (HUG), Switzerland. [13]Faculty of Medicine Siriraj Hospital, Department of Dermatology , Mahidol University , Bangkok , Thailand. [14]SDC - The Skin Doctors' Center, Trieste, Italy. [15]Dermatology and Cutaneous Surgery, University of Miami School of Medicine, 1475 NW 12th Ave., Miami, FL, 33136, USA. [16]Department of Dermatology, Venereology and Allergy, University of Leipzig. [17]University of Côte d'Azur, University Hospital Nice, Department of Dermatology, Nice, France. [18]University of Côte d'Azur, Centre Méditéranéen de Médecine Moléculaire (C3M), INSERM U1065, team 12, Nice, France. [19]Department of Epidemiology and Biostatistics, Amsterdam Public Health research institute, Amsterdam UMC, The Netherlands. [20]Verner Clinic, Tel Aviv, Israel.

For peer review only

Corresponding author

Frederike Fransen

Department of Dermatology, Amsterdam UMC

Meibergdreef 9

1105 AZ Amsterdam

Phone: +31 20 56 67 792

E-mail: frederikefransen@gmail.com

Abstract word count:	245
Main text word count:	3039
Table count:	2
Figure count:	1
Supplementary Files:	4
References:	26

Funding sources statement:
This manuscript presents independent research that has been supported by a research grant from the European Academy of Dermatology and Venereology (EADV Project proposal reference number 2017-035).

For peer review only - http://bmjopen.bmj.com/site/about/guidelines.xhtml

ABSTRACT

Introduction: While laser technology has expanded the armamentarium of treatment for various skin diseases during the past years, heterogeneity in study outcomes hampers comparability and appropriate evidence synthesis. Part of these issues can be addressed by developing a generic outcome set. Using the Delphi method, this study aims to seek consensus between key stakeholders on relevant generic outcomes (*what* to measure) for implementation in the international registry on Laser trEAtments in Dermatology (LEAD). The registry is focused on collecting research data on various laser treatments for skin disorders.

Methods and analysis: By reviewing the literature and involvement of key stakeholder groups and adult patients in need or after laser surgery and health professionals, a preliminary list of outcomes will be generated and categorized into domains. Using these outcomes, an international three-round Delphi study will be performed to rate the importance of outcomes in the selection of a generic outcome set. Participants are allowed to provide new outcomes to the prelimary list for revisions during the first Delphi round. Finally, results will be discussed during a consensus meeting to agree on generic outcomes to be used in the LEAD Registry.

Ethics and Dissemination: An ethics approval was not applicable (W19_290 # 18.336). The study is registered with the CS-COUSIN (Cochrane Skin Core OUtcome Set INitiative) and the Core Outcome Measures in Effectiveness Trials (COMET) initiative. Procedures will be conducted according to the Declaration of Helsinki. The findings will be disseminated through peer-reviewed publications and conference presentations.

For peer review only

Keywords: Laser Therapy, Dermatology, Consensus study, Delphi study, Disease registry, Generic Outcome Set

ARTICLE SUMMARY

Strengths and limitations of this study

- This protocol outlines the first international consensus effort to develop a generic outcome set for use in the international LEAD laser registry.
- With advances in laser technology, considering outcomes of importance (*what* to measure) to patients and health professionals is crucial.
- A comprehensive systematic review will explore which outcomes are used and reported in existing studies on laser treatments.
- The Delphi procedure requires three survey rounds and involves a large group of stakeholders across various disciplines and geographical areas including patients, reflecting different viewpoints.

For peer review only

INTRODUCTION

During the past decades, modifications in laser technology have further widened its scope and greatly expanded the cutaneous laser surgeon's armamentarium [1,2]. Today, there are many medical indications in dermatology, encompassing vascular, pigmented, inflammatory, metabolic or infectious lesions, benign tumours, scars, and hair follicle- related skin conditions that are regularly - and sometimes exclusively - treated with lasers [1–3]. Many of these disorders meet the criteria of an orphan disease.

The diversity in laser devices and the spectrum of medical indications pose unique research challenges for clinical decision-making in laser therapy. Because most laser physicians are not exposed to large numbers of patients receiving laser treatments for uncommon indications, knowledge on the most effective laser treatment, including safety and used regimen, is unclear. The current evidence for most of these specific skin conditions is sporadic at best, consisting mostly of case reports and case series and only a very small number of randomized controlled trials (RCTs) [4,5]. Moreover, most often only isolated successes are reported while cases that failed to respond are not published, leading to publication bias [6]. Another issue hampering evidence synthesis is heterogeneity of outcome definition, measurement and reporting in laser research. Patient-reported outcomes (PROs), such as 'patient experience of laser treatments' and 'health-related quality of life', are often not reported and together with selective outcome reporting in laser research, it is all a serious threat to comparative effectiveness research as it limits the ability to compare, contrast, and combine individual studies [7,8]. As a result, this hampers to draw meaningful conclusions and guidance to inform clinical decision-making [9,10].

For peer review only

For peer review only - http://bmjopen.bmj.com/site/about/guidelines.xhtml

To overcome this issue in the field of laser dermatology, the development of the International Laser Treatment (LEAD) Registry has been proposed to initiate collaborative data pooling of a wide range of skin disorders. The development of a registry may be the key to the lack of solid evidence for laser treatments in dermatology, however, well-defined standardized and generic outcomes are required for its establishment.

To address the variations in outcome reporting, organizations such as the Core Outcome Measures in Effectiveness Trials Initiative (COMET) bring together researchers interested in developing a standardized set of core outcomes in various health-related fields [11]. A core outcome set (COS) is defined as an agreed minimum set of outcomes that should be measured and reported in all clinical trials for a specific health condition, including methods used to measure these core outcomes[10,12]. Throughout this report, the definition of "outcome" refers to a single construct that can be measured as a standalone item (e.g. 'erythema'), while the term "outcome domain" or "domain" is an umbrella term for a group of associated outcomes (e.g. 'signs as assessed by physician'). Furthermore, the outcome instrument refers to how the outcomes are measured. Although a COS is recommended for clinical trials, they can also be developed for routine clinical practice, and for registries [10,12]. In 2015, the international, multidisciplinary working group, the Cochrane Skin Group- Core OUtcome Set INitiative (CS-COUSIN) has been established [13]. The organization supports dermatology-specific initiatives to develop and implement a COS by building upon experiences of the Harmonizing Outcome Measures for Eczema (HOME) initiative, which developed a roadmap to guide the process of COS development and implementation [14]. Currently, 17 COS initiatives have been supported by CS-COUSIN in dermatology. These projects involve 26 different skin diseases, such as acne, atopic eczema, hidradenitis suppurativa, melanoma, nail psoriasis,

For peer review only - http://bmjopen.bmj.com/site/about/guidelines.xhtml

rosacea, and vitiligo [11,15]. However, with hundreds of different and mostly unrelated dermatoses that are treated with lasers in the field of laser dermatology, the need for a generic outcome set (GOS) is commanding. Therefore we focus on developing a GOS (*what* to measure) for the purpose of the LEAD registry. The GOS is intended to be applied for the assessment of various, unrelated skin diseases that are treated with different types of lasers.

In summary, there is an urgency of using the same generic outcomes in laser therapy. Hence, establishing consensus on the relevant outcomes for the LEAD registry will promote clinical researchers to use outcomes chosen by consensus that are relevant to patients and clinicians. The use of generic outcomes support data synthesis for many diseases in dermatology. The protocol outlines the context, scope and methods for the development of a GOS to be implemented in the LEAD registry.

Aims and objectives

Aim

The aim of this study is to reach consensus between various stakeholders on generic outcomes relevant for the LEAD registry.

Objectives

Our study objectives are:

1. To identify outcomes that have previously been used and reported in RCTs, cohort studies, case-control studies and case series from a literature review and classify these outcomes into domains according to the COMET taxonomy;

2. To reach consensus between stakeholders on the outcomes of a GOS to be implemented in the LEAD registry.

For peer review only - http://bmjopen.bmj.com/site/about/guidelines.xhtml

Scope and applicability of outcomes

The registry is envisioned to suit all types of laser interventions for skin disorders in dermatology including vascular, pigmented or inflammatory lesions , benign tumours, scars, and hair follicle-related skin conditions treated with lasers. The GOS is intended for use in the LEAD registry, with the focus on prospectively recording the effectiveness and safety of cutaneous non cosmetic laser interventions. Therefore we excluded laser assisted drug delivery, low laser level therapy, body- contouring, skin tightening, hair removal, rejuvenation and anti-aging procedures. Furthermore, because of the distinctive mode of action and use in daily clinical practice, laser assisted drug delivery, low laser level therapy and laser procedures for (leg) veins were excluded.

METHODS AND ANALYSIS

Research group

The steering committee (FF, PS, AW, MA, AB, PB, IH, MH, LH ,KK, TK, HL, WM, LM, KN, UP, TP, CP, IV) provide input at critical points of the study such as protocol development, stakeholder recruitment, consensus process and the consensus meeting. Three members of the steering committee (FF,PS,AW) coordinate the overall project, ensure methodological quality of the project and make key decisions. All members of the steering committee will participate in the Delphi procedure as well as in the final consensus meeting. The steering committee has representatives from The Netherlands, Denmark, Egypt, France, Germany, Israel, Italy, Japan, Spain, Switzerland, Thailand and USA, with extensive expertise in various laser treatments, outcomes research and clinical research. A list of all members of the steering committee is given in supplementary file 1.

For peer review only - http://bmjopen.bmj.com/site/about/guidelines.xhtml

For peer review only

Study design

Figure 1 provides a brief overview of the stepwise approach with different research methods. The study consists of the following two phases:

Phase 1: Identification of potential outcomes important in laser treatments by means of a

1. A systematic review to form the preliminary list of outcomes for the Delphi survey

2. Classification of outcomes into domains according to the COMET taxonomy [19]

Phase 2: A consensus process involving key stakeholders who are able to suggest additional outcomes during the first round and who will rate the importance of outcome for reaching consensus on the GOS by means of a

1. Three-round Delphi survey.

2. Expert consensus meeting. attended by representatives of all stakeholder groups.

This study is registered with the CS-COUSIN and COMET initiative [11,16]. Results of the consensus study will be reported according to the Core Outcome Set-STAndards for Reporting (COS-STAR) [17].

For peer review only - http://bmjopen.bmj.com/site/about/guidelines.xhtml

Phase 1: Identification of potential outcomes and domains

Phase 1.1: Systematic literature review

The first phase of the study is to identify which outcomes should be measured and reported in a registry on laser treatments for skin disorders (what to measure: the GOS, see definitions in supplementary file 2). A SR will be performed to explore existing outcomes that are used in laser studies. According to the COMET guidelines [18], searches will be performed in the following database: MEDLINE and EMBASE. Articles between January 2013 and December 2017 will be retrieved. The electronic search strategy is detailed in supplementary file 3. A recent 5-year time period has been selected for the search so that outcomes extracted represent the practice of present-day laser research. The inclusion and exclusion criteria are presented in Table 1. Two reviewers will select articles and extract the data independently. Disagreement will be resolved by discussion and by consulting a third review author if necessary. The following data will be extracted from the selected articles in data extraction tables : authors, years of publication, country, cutaneous indications for treatment and type of laser treatments. We will assess what outcomes and outcome measurement instrument are used, consistency in outcomes, number of times an outcome was used, consistency in classification used.

For peer review only - http://bmjopen.bmj.com/site/about/guidelines.xhtml

251 **Table 1** Inclusion and exclusion criteria for literature review

252

	Inclusion criteria	Exclusion criteria
Patient population and indication	Studies including patients age 18 and older with vascular, pigmented, inflammatory, metabolic or infectious lesions, benign tumours and hair follicle-related skin conditions treated with lasers	Non-humans flebological skin conditions Laser assisted drug delivery, low laser level therapy, body-contouring, skin tightening, hair removal, rejuvenation and anti-aging
Study design	RCTs, cohort studies, case-control studies, case series	In vitro studies, systematic reviews, abstracts and expert opinions, case reports
Intervention	Any type of laser treatment for vascular, pigmented or inflammatory lesions, benign tumours, and hair follicle-related skin conditions.	Laser assisted drug delivery, low laser level therapy, laser therapy for leg veins and cosmetic interventions (see scope of outcomes)
Outcomes		Non-clinical outcomes e.g. biochemical outcomes, imaging, confocal laser, histology
Publication	All studies are conducted between 2013-2017	

253

254

255

256

257

258

259

260

261

For peer review only - http://bmjopen.bmj.com/site/about/guidelines.xhtml

Phase 1.2: Classification of outcomes into domains

Subsequently, data will be classified according to the standardized taxonomy for outcomes proposed by the COMET initiative [19]. This taxonomy encompasses 38 domains within 5 core areas: mortality/survival; physiological/clinical; life impact; resource use; adverse events.

Outcomes and their classification in domains will be discussed with three members (FF, PS, AW) of the steering committee. The preliminary list of outcomes classified to domains will be included in the consensus process.

Phase 2: Consensus process

Phase 2.1: Delphi procedure

For investigating crucial outcomes in context of the LEAD registry, a Delphi study will be conducted. The Delphi is based on a structured process for gathering and condensing knowledge from key stakeholder groups by means of 3 rounds with a series of questionnaires [20]. The procedure will consist of three online rounds (Figure 1).

Participants

The involvement of a variety of stakeholders is a key part for the identification of outcomes and strongly recommended by methodologists [21].

The following representatives from four international key stakeholder groups are involved in the process of reaching consensus on outcomes:

For peer review only

1. Patients of age 18 with vascular, pigmented, inflammatory, metabolic or infectious lesions, benign tumours and hair follicle-related skin conditions treated by lasers.

2. Patient representatives involved in patient associations that raise awareness on the impact of vascular, pigmented, inflammatory, metabolic or infectious lesions, benign tumours and hair follicle-related skin conditions.

3. Health care professionals – Laser experts who treat patients with vascular, pigmented or inflammatory, metabolic or infectious lesions, benign tumours, hair follicle-related skin conditions and who are involved in research on laser treatments.

4. Health care professionals –General physicians who treat patients with dermatological indications.

Panel size and recruitment

There is no robust guidance for calculating the number of participants needed for a Delphi study and expectations are based on COMET Initiative guidelines and previous literature [16,22,23]. As there are various stakeholder groups involved in the Delphi procedure, we will recruit as many international representatives as possible from each group. All potential participants will be invited with a letter explaining the aims and details of the study and the rationale and importance of completing the entire Delphi process. Respondents who agree to take part will be assigned a unique identification number. Furthermore, each member of the steering committee will be asked to cascade the link of the survey to 3 other physicians in their network. Patients and patient representatives will be recruited from national and international support groups for skin diseases treated with lasers and can be found in supplemental file 4**.** In addition, laser experts from the

For peer review only - http://bmjopen.bmj.com/site/about/guidelines.xhtml

steering committee will be asked to recruit 3 patients with different skin conditions treated with lasers in their center. To make sure that we involve skin diseases of different categories, laser experts will indicate the diagnosis of the patients that are recruited. By sending the survey invitation to experts and patient support groups from different continents, we aim to reflect a broad range of patients and health professionals with diverse backgrounds and experiences. For each round, the number of participants invited and those who completed the surveys will be documented. The participants will have 3 weeks to complete each round. We will send personal reminder emails to those who did not respond after 7 and 14 days to increase the response rate.

Delphi survey

Participants will be divided into a group of patient and a group of health professional , leading to separate scoring of outcomes. All participants will be asked to rate the importance of each of the outcomes using the GRADE (Grading of Recommendations Assessment, Development and Evaluations) approach. The scale will range from 1 to 9 and will be categorized as follows: 1–3 'not important'; 4–6 'important but not critical'; and 7–9 'critical' [24,25]. If participants feel unable to rate or provide feedback they can select 'unable to score'.

Delphi rounds

Delphi round 1 .

During the first round of the Delphi survey, baseline characteristics (age, gender, country of practice) will be obtained from all participants. Patients will be asked for their medical indication and type of laser treatment, and whether any complications have occurred during treatment.

For peer review only - http://bmjopen.bmj.com/site/about/guidelines.xhtml

326 Health professionals will be asked their specialty (laser dermatology, general dermatology or
327 other), workplace (academic, teaching hospital or non-teaching hospital) and years in practice.
328 Next, participants will be asked to score listed outcomes and will have the option to suggest any
329 additional outcomes that are not yet presented in the preliminary list.

330 *Delphi round 2 and 3*

331 In the second and third Delphi rounds, all participants will receive feedback on the scores of the
332 previous round in both the patient and the health professional group. The outcomes from the
333 previous rounds will be presented with the median scores from each stakeholder group
334 combined with a histogram showing the scoring distribution. Subsequently, participants will be
335 asked to score all outcomes for which consensus has not been reached, in the same manner as
336 in the first Delphi round. Outcomes for which there was only consensus within a single
337 stakeholder group will also be shown to the other stakeholder group to evaluate whether
338 consensus can be achieved in both stakeholder groups.

339

340 **Definition of consensus**

341 The definition of consensus is presented in Table 2. 'Consensus in' is defined as approval of the
342 outcome by the vast majority (70 %) of all stakeholder groups that score 7, 8, or 9 with fewer
343 than the minority (15 %) of panelists scoring 1–3. On the contrary, 'consensus out' is defined as
344 70% or more of all stakeholder groups scoring as 1 to 3 and less than 15% scoring as 7 to 9 [12].
345 After three e-Delphi rounds, outcomes will be classified as 'consensus in' (consensus on the
346 importance of the outcome), 'consensus out' (no consensus on the importance, or consensus on
347 nonimportance) or 'no consensus' (consensus on the importance in only one or or no consensus).

For peer review only - http://bmjopen.bmj.com/site/about/guidelines.xhtml

Table 2: Definitions of consensus for identifying generic outcomes for the LEAD registry

Consensus category	Clarification	Definition
Consensus in	Outcome should be included in the registry	70% of stakeholder groups scoring as 7 to 9 and < 15% of stakeholder groups scoring as 1 to 3
Consensus out	Outcome should not be included in the registry	70% or more of stakeholder groups scoring as 1 to 3 and < 15% of stakeholder groups scoring as 7 to 9
No consensus	Hesitation about relevance of outcome to be included in the registry	Anything other

Phase 2.2: Determination of the GOS during the expert consensus meeting

In case complete consensus is reached in the Delphi procedure on the outcomes of the GOS , no formal consensus meeting will be organized. However, the results of the Delphi will be discussed with three members of the steering committee (FF. PS, AW) to check misconceptions in the Delphi method and to safeguard a well-defined GOS. For outcomes for which consensus definition during the Delphi has not been reached, we invite 15 participants from across all stakeholder groups to participate in an online expert consensus meeting within 2 months after the close of round 3. The primary goal of the meeting is discussing the 'no consensus' outcomes. Consensus results from the Delphi can be reversed in this meeting if reasons are very strong and clear.

For peer review only - http://bmjopen.bmj.com/site/about/guidelines.xhtml

Patient and public involvement

Patient and public were not involved in the development of this study protocol. However, patients will be involved and included within the Delphi procedure as expert group. Consensus methodology will ensure that the opinions and preferences of patients will be given the same weighting as those of the laser experts and health professionals. Furthermore, patients will participate in the final consensus meeting. We disseminate the main results to study participants and patients by email which will include a copy of the final outcomes of the GOS. In addition, where approval has been given, participants (including members of the public) will be named as contributors in the acknowledgments section.

DISCUSSION

By the end of this study, we hope to reach consensus on a GOS that could be implemented in an international registry with a research focus, that collects data of rare skin diseases treated by lasers. Analysis of registry data provides insight into effectiveness and safety of different laser treatments across many skin diseases, laser centers and countries.

There are several strengths using the Delphi method for this study. First, the Delphi method allows to recruit a large number of laser experts, physicians and patients from diverse regions globally. The diversity in the experts' backgrounds and expertise ensures maximum impact of the results. Secondly, the Delphi method is the accurate tool in consensus processes in various

For peer review only

stakeholder groups as individuals are able to express their own opinions and feedback can be provided in a controlled anonymous way. This means that there is room for individual disagreement but also consideration of the answers given by other individuals and stakeholder groups as a whole. However, there are also limitations of the Delphi method. Results are dependent upon the composition of the participants. There is a risk of relative uneven representations among patients, but also health professionals. Especially, when focusing on a specific group of rare skin diseases, selection bias could result in insufficient representation of other skin disorders. We request health professionals of the steering committee to recruit patients with 3 different skin disorders. Through this method, we hope to ensure that all subgroups including vascular, pigmented, metabolic, inflammatory lesions, benign tumours and hair follicle-related skin conditions, will be adequately involved. For patients it might be a barrier to imagine what is important to be included in a registry for a broad range of diseases, rather than one disease that is important to themselves. We will stress the importance of agreeing on a GOS for all diseases in each round of the Delphi survey and consensus meetings. Photographs will be included to illustrate the variety of skin disorders that are involved. To provide the highest possible input we will extend our invitation to take part in the Delphi survey to patients and health professionals in Africa, Asia, South-America, Australia, in addition to Europe and North-America. With support from all panel members we hope to ensure that the LEAD registry will be internationally relevant, accepted and ready to use.

For peer review only - http://bmjopen.bmj.com/site/about/guidelines.xhtml

Trial status

The identification of generic outcomes for registry use is ongoing and in the initial phase. A systematic review has been performed to explore current outcomes used and reported in laser dermatology. We are currently preparing to recruit participants for the Delphi study. The generic outcomes s are expected to be implemented in the laser registry in 2020.

ETHICS AND DISSEMINATION

The medical research ethics committee of the Academic Medical Center Amsterdam confirmed that the Dutch Medical Research Involving Human Subjects Act does not apply to this study (W19_290 # 18.336) and that complete approval of this study by the committee is not necessary. All participants involved in the Delphi study will be asked for their consent before taking part. All procedures will be conducted according to the Declaration of Helsinki. All results from the consensus study will be reported in peer-reviewed indexed journals. The data will be presented at conferences chosen to reach a wide range of knowledge users.

Abbreviations

COMET: Core Outcome Measures for Effectiveness Trials; GOS: Generic Outcome Set; CSG-COUSIN: Cochrane Skin Group—Core Outcome Set Initiative; COSMIN: COnsensus-based Standards for the selection of health Measurement Instruments; GOS: Generic Outcome Set; GRADE: Grading of Recommendations Assessment; LEAD registry: Laser TrEAtment Dermatology registry; RCT: Randomized controlled trial.

Contributors

FF initiated the protocol, designed the study, wrote the manuscript and reviewed it for

For peer review only - http://bmjopen.bmj.com/site/about/guidelines.xhtml

For peer review only

important intellectual content. PS contributed significantly to the study design and reviewed the manuscript for important intellectual content. CP contributed to the study design and reviewed the manuscript for important intellectual content. AW initiated the protocol, designed the study and reviewed it for important intellectual content. All authors (FF, PS, AW, MA, AB, PB, IH, MH, LH ,KK, TK, HL, WM, LM, KN, UP, TP, CP, IV) read and approved the final manuscript.

Acknowledgements

We are grateful to Jan Kottner of the CS-COUSIN methods group for providing advice for methodological issues during the protocol development. We acknowledge Marjolein van Kessel as patient advocate of Naevus International for her support in preparing the Delphi rounds.

Supplementary Files

1. List of members of the LEAD registry steering committee
2. A glossary on the definitions of terminology
3. Search strategy for the systematic review of literature
4. A list of invited patient support groups for the Delphi survey

REFERENCES

1 Tanzi EL, Lupton JR, Alster TS. Lasers in dermatology: Four decades of progress. J. Am. Acad. Dermatol. 2003;**49**:1–31. doi:10.1067/mjd.2003.582

2 Husain Z, Alster TS. The role of lasers and intense pulsed light technology in dermatology. Clin. Cosmet. Investig. Dermatol. 2016;**9**:29–40. doi:10.2147/CCID.S69106

3 Houk LD, Humphreys T. Masers to magic bullets: an updated history of lasers in dermatology. *Clin Dermatol* 2007;**25**:434–42. doi:10.1016/j.clindermatol.2007.05.004

4 U.S. National Institutes of Health. ClinicalTrials.gov. clinicaltrials.gov. 2013.http://clinicaltrials.gov/ (accessed 28 Mar 2018).

For peer review only - http://bmjopen.bmj.com/site/about/guidelines.xhtml

5 Fransen F, Tio D, Prinsen CAC, *et al.* A Systematic Review of Outcome Reporting in Laser Treatments for Dermatological Diseases. *J Eur Acad Dermatology Venereol* Published Online First: 2019. doi:10.1111/jdv.15928

6 Atakpo P, Vassar M. Publication bias in dermatology systematic reviews and meta-analyses. *J Dermatol Sci* Published Online First: 2016. doi:10.1016/j.jdermsci.2016.02.005

7 Snyder CF, Aaronson NK, Choucair AK, *et al.* Implementing patient-reported outcomes assessment in clinical practice: A review of the options and considerations. Qual. Life Res. 2012. doi:10.1007/s11136-011-0054-x

8 Prinsen CAC, Mokkink LB, Bouter LM, *et al.* COSMIN guideline for systematic reviews of patient-reported outcome measures. *Qual Life Res* Published Online First: 2018. doi:10.1007/s11136-018-1798-3

9 Kirkham JJ, Gorst S, Altman DG, *et al.* COS-STAR: A reporting guideline for studies developing core outcome sets (protocol). *Trials* 2015;**16**. doi:10.1186/s13063-015-0913-9

10 Clarke M. Standardising outcomes for clinical trials and systematic reviews. Trials. 2007;**8**. doi:10.1186/1745-6215-8-39

11 http://www.comet-initiative.org/

12 Williamson PR, Altman DG, Blazeby JM, *et al.* Developing core outcome sets for clinical trials: Issues to consider. *Trials* Published Online First: 2012. doi:10.1186/1745-6215-13-132

13 Schmitt J, Deckert S, Alam M, *et al.* Report from the kick-off meeting of the Cochrane Skin Group Core Outcome Set Initiative (CSG-COUSIN). In: *British Journal of Dermatology*. 2016. 287–95. doi:10.1111/bjd.14337

14 Schmitt J, Apfelbacher C, Spuls PI, *et al.* The Harmonizing Outcome Measures for Eczema (HOME) Roadmap: A Methodological Framework to Develop Core Sets of Outcome Measurements in Dermatology. *J Invest Dermatol* Published Online First: 2015. doi:10.1038/jid.2014.320

15 Prinsen CAC, Spuls PI, Kottner J, *et al.* Navigating the landscape of core outcome set development in dermatology. *J Am Acad Dermatol* Published Online First: 2019. doi:10.1016/j.jaad.2019.03.009

16 Williamson P. The comet initiative. *Trials* 2013;**0**:68DUMMY.http://ovidsp.ovid.com/ovidweb.cgi?T=JS&CSC=Y&NEWS=N&PAGE=fulltext&D=emed13&AN=71799862%5Cnhttp://oxfordsfx.hosted.exlibrisgroup.com/oxford?sid=OVID:embase&id=pmid:&id=doi:&issn=1745-6215&isbn=&volume=14&issue=&spage=68DUMMY&pages=68DUMMY&date=2013&title=

17 Kirkham JJ, Gorst S, Altman DG, *et al.* Core Outcome Set–STAndards for Reporting: The COS-STAR Statement. *PLoS Med* 2016;**13**. doi:10.1371/journal.pmed.1002148

18 Prinsen CAC, Vohra S, Rose MR, *et al.* Core Outcome Measures in Effectiveness Trials (COMET) initiative: Protocol for an international Delphi study to achieve consensus on how to select outcome measurement instruments for outcomes included in a 'core outcome set'. *Trials* 2014;**15**. doi:10.1186/1745-6215-15-247

19 Dodd S, Clarke M, Becker L, *et al.* A taxonomy has been developed for outcomes in medical research to help improve knowledge discovery. *J Clin Epidemiol* 2018;**96**:84–92. doi:10.1016/j.jclinepi.2017.12.020

20 Jones J, Hunter D. Consensus methods for medical and health services research. *Br Med J* Published Online First: 1995. doi:10.2164/jandrol.111.015065

For peer review only

For peer review only - http://bmjopen.bmj.com/site/about/guidelines.xhtml

21 Harman NL, Bruce IA, Kirkham JJ, *et al.* The importance of integration of stakeholder views in core outcome set development: Otitis Media with Effusion in children with cleft palate. *PLoS One* Published Online First: 2015. doi:10.1371/journal.pone.0129514

22 Young A, Brookes S, Rumsey N, *et al.* Agreement on what to measure in randomised controlled trials in burn care: Study protocol for the development of a core outcome set. *BMJ Open* 2017;**7**. doi:10.1136/bmjopen-2017-017267

23 Schlessinger DI, Iyengar S, Yanes AF, *et al.* Development of a core outcome set for clinical trials in squamous cell carcinoma: Study protocol for a systematic review of the literature and identification of a core outcome set using a Delphi survey. *Trials* 2017;**18**. doi:10.1186/s13063-017-2069-2

24 Balshem H, Helfand M, Schünemann HJ, *et al.* GRADE guidelines: 3. Rating the quality of evidence. *J Clin Epidemiol* Published Online First: 2011. doi:10.1016/j.jclinepi.2010.07.015

25 The Grading of Recommendations Assessment Development and Evaluation. GRADE working group. *Grade* 2014.

Figure legends

Figure 1: Flow diagram outlining the development of a generic outcome set for the LEAD registry.
Preparatory stages and process of consensus for relevant generic outcomes are summarized.

For peer review only

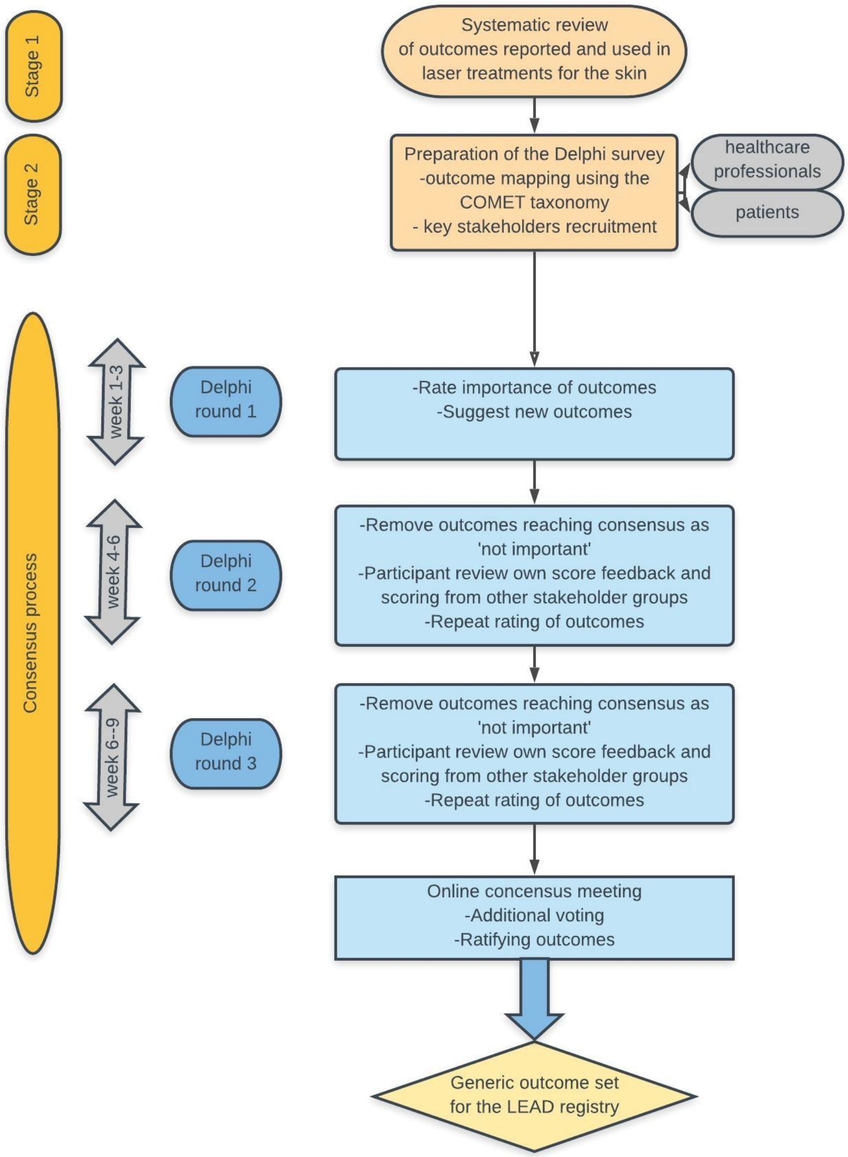

Flow diagram outlining the development of a generic outcome set for the LEAD registry.

206x277mm (160 x 160 DPI)

Amsterdam UMC
Universitair Medische Centra

Dr. Adrian Aldcroft
London, UK
Editor-in-Chief
BMJ Open

December 21 th, 2019

Dear Editor-in-Chief Dr. Adrian Aldcroft,

Re: Manuscript ID bmjopen-2018-025361 (previously) entitled "Study Protocol for the Identification of Outcomes in Skin Laser Therapy: A Starting Point for the European Laser Treatment Registry"

We would like to thank you for considering the above titled paper for publication in BMJ open.

We have made the major and necessary revisions. An itemized response to each suggested revision is enclosed. We assert that each named author has approved the final version of this enclosed manuscript.

We hope that this clarification and revision persuade you to accept our submission. Thank you for your consideration of our revised manuscript, we would be glad to respond to any further questions and comments that you may have.

We look forward to hearing from you.

On behalf of the authors, yours sincerely,

Frederike Fransen, MD
Department of Dermatology, Amsterdam UMC
Phone: +31 6 51 99 38 31
Email: frederikefransen@gmail.com/f.fransen@amc.uva.nl

For peer review only

Dear Reviewers,

We thank you for taking the time to carefully read our manuscript that is currently entitled " A generic outcome set for the international registry on Laser TrEAtments in Dermatology (LEAD): a protocol for a Delphi study to achieve consensus on what to measure " and for the valuable comments you have provided. Please find below our detailed response to each of the comments..

On behalf of all co-authors,

Yours sincerely,

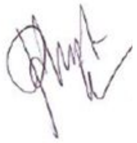

Frederike Fransen, MD
Department of Dermatology, Amsterdam UMC
Phone: +31 6 51 99 38 31
Email: frederikefransen@gmail.com/f.fransen@amc.uva.nl

For peer review only

For peer review only - http://bmjopen.bmj.com/site/about/guidelines.xhtml

<u>Editor Comments to Author:</u>

We would like to thank the Editor for the feedback and we appreciate the valid assessment of our protocol.

Please include the study design in title.

We changed the title of the protocol, including the study design (Delphi consensus).

The initial title 'Study Protocol for the Identification of Outcomes in Skin Laser Therapy: A Starting Point for the European Laser Treatment Registry' has been changed to 'A generic outcome set for the international registry on Laser TrEAtments in Dermatology (LEAD): a protocol for a Delphi study to achieve consensus on what to measure'

Authors must include a statement in the methods section of the manuscript under the sub-heading 'Patient and Public Involvement'. This should provide a brief response to the following questions:

How was the development of the research question and outcome measures informed by patients' priorities, experience, and preferences?

How did you involve patients in the design of this study?

Were patients involved in the recruitment to and conduct of the study?

How will the results be disseminated to study participants?

For randomised controlled trials, was the burden of the intervention assessed by patients themselves?

We included a statement in the methods section of the manuscript under the sub-heading 'Patient and Public involvement' covering the responses to the questions above.

In the methods section we added, line 362-370:

"Patient and public were not involved in the development of this study protocol. However, patients will be involved and included within the Delphi procedure as expert group. Consensus methodology will ensure that the opinions and preferences of patients will be given the same weighting as those of the other laser experts and health professionals. Furthermore, patients will participate in the final consensus meeting. To make sure the Delphi questionnaire is understandable and has no ambiguities, we received input from a patient representative. Also, the questionnaire is tested by a group of patients and health professionals before the start of the Delphi study. Part of the Delphi study is giving feedback to all its participants after each round; this will also be done with the final study results. We intend to disseminate the main results to study participants and patients. On completion of the Delphi study, all participants (experts, health professionals and patients) will be sent an email with a copy of the final outcomes for the LEAD . In addition, where consent has been given, participants (including members of the public) will be named as contributors in the results publication."

Patient advisers should also be thanked in the contributorship statement/acknowledgements.

We added the patient advocate to our acknowledgements. See line 435-438 "We acknowledge Marjolein van Kessel as patient advocate of Naevus International for her support in drafting the protocol."

If patients and or public were not involved please state this.

Patients are involved in the Delphi consensus study, however, as reported in methods section line 369, they are not involved in drafting the protocol.

Please provide a more detailed contributorship statement. It needs to mention all the names/initials of authors along with their specific contribution/participation for the article.

A more detailed contributorship statement including names and initials of authors has been included. Section Contributors, line 420-427.

Please provide another copy of your figures with better qualities and please ensure that Figures are of better quality or not pixelated when zooming in. NOTE: They can be in TIFF or JPG format and make sure that they have a resolution of at least 300 dpi and at least 90mm x 90m of width. Figures in PDF, DOCUMENT, EXCEL and POWER POINT format are not acceptable.

The figure has been changed with details of the Delphi study with a better quality. We will provide the figure in JPG format with a resolution of a least 300 dpi.

For peer review only - http://bmjopen.bmj.com/site/about/guidelines.xhtml

1. Reviewer: 1
Reviewer Name: Travis Blalock
Institution and Country: Emory University SOM

We would like to thank the reviewer for the suggestions and we appreciate the valid assessment of our protocol.

Line 86: you cannot say that lasers are the treatment of choice for these entities... surely great treatments, but "treatment of choice" is incorrect for tumors and others.
We agree with the reviewer that laser treatments are not in all cases the treatment of choice, especially in cases focusing on skin tumors. We have made the changes accordingly. Introduction section, manuscript page 5, line 115-119, words: "Today, there are many medical indications in dermatology, encompassing vascular, pigmented, inflammatory, metabolic or infectious lesions, benign tumours, scars, and hair follicle- related skin conditions that are regularly - and sometimes exclusively - treated with lasers [1–3]."

Line 149: delete "a long list of"
"a long list of" has been deleted.

Line 152: Is the goal 'expert' or 'provider?'
Our goals is here to create a list of outcomes from a laser expert perspective.

Reviewer: 2
Reviewer Name: Dr Freedom Gumedze
Institution and Country: University of Cape Town, Department of Statistical Sciences, P D Hahn Building, Room 6.63, Private Bag, Rondebosch 7701, South Africa

We would like to thank for the recommendations of the reviewer and we appreciate the valid assessment.

Please leave your comments for the authors below
Page 1, Table 1: Under study design would registries be included? If no, why not?
To our knowledge, there have not been any registries focusing exclusively on uncommon dermatological diseases that are treated by lasers.

Page 1, Table 1: Can the authors justify the study period of 2013-2017. Would this include studies that are ongoing or completed after 2017 with preliminary findings or final results?
We started our study and protocol in the beginning of 2018. This means that also studies that are completed after 2017 with final results are included in the review. Our goal is to show the outcomes used in the past years. Due to time limitations it is not our intention to create the most up-to-date (all articles of 2018 and 2019) version.

Page 6, line 104: Rephrase as [RCTs) [10,11]. Here and everywhere in the text fullstop must be inserted after the references.
Thank you for mentioning the in-text citations details. We inserted full stops after all references throughout the manuscript.

Page 25, Figure 1: Can the authors give an indication of the duration of each phase or Delphi round and the entire duration of the study.

Figure 1 has been changed to provide an indication of the duration of each Delphi round (3 weeks). This has also been reported in the manuscript

Page 14, Methods section, 'Panel size and recruitment) Line 309-310, words: "Participants will have 3 weeks to complete each round, depending on the response rate. Furthermore, we will send personal reminder emails to those who did not respond after 7 and 14 days to increase the response rate."

The expectations of the entire duration of the study is indicated in the manuscript text, see below.

For peer review only - http://bmjopen.bmj.com/site/about/guidelines.xhtml

Section 'Trial status',page 19, line 400, words "The generic outcomes are expected to be implemented in the laser registry by the beginning of 2020".

<u>Reviewer: 3</u>
Reviewer Name: Jennifer Zuccaro
Institution and Country: Hospital for Sick Children, Division of Plastic and Reconstructive Surgery, Toronto, Canada

Thank you for supporting us by reviewing the manuscript. We appreciate the dedication and efforts for correcting the manuscript as well.

General Comments:
This is an important protocol that highlights the need for standardized outcome reporting in the field of laser medicine. In addition to the comments below, the following points should be considered:

Provide further background and justification for why it is crucial that outcomes for laser therapy be identified for patients with skin conditions specifically as opposed to other indications.

In accordance with the suggestions of the reviewer, we now adjusted the introduction section of the manuscript and clearly explained the importance of standardized generic outcomes for dermatological diseases treated with lasers in context of a core outcome set for specific skin diseases.
Introduction section: line 152" In 2015, the international, multidisciplinary working group, the Cochrane Skin Group- Core Outcome Set Initiative (CS-COUSIN) has been established" until line 161-164 "Therefore we focus on developing a generic set of outcomes (GOS) for the purpose of the LEAD registry. The GOS is intended to be applied to the assessment of various, unrelated skin diseases that are treated with different types of lasers."

Provide further information for how the e-Delphi will actually run and clarify how long each round will take. Please specify if the length of each round will be determined by time or by participant saturation and when you expect the process to be complete.

We changed figure 1 and clarified the length of each Delphi round, determined by time (3 weeks for each round). The expectations of the entire duration of the study is indicated in the manuscript text. The expectations of the entire duration of the study is indicated in the manuscript text, see below.

Section 'Trial status',page 19, line 404, words "The generic outcomes are expected to be implemented in the laser registry by the beginning of 2020".

All spelling and grammar corrections are included on the attached protocol.

Thank you for supporting us with the corrections. We appreciate the dedication and efforts for reviewing the manuscript. We corrected the spelling and grammar.

1. Line 59/60: Please clarify what meant by "rapidly evolving laser treatments." Specifically, what aspect is evolving?

We mean with 'rapidly evolving laser treatments' that refinements in laser technology have progressed so rapidly during the past decade that successful treatment of many cutaneous can be achieved. We changed the part of the sentence to "<u>With modifications in laser technology</u>, considering outcomes of importance (what to measure) to patients and health professionals is crucial."
Page 4, Article summary 'Strengths and limitations of this study', line 102.

2. Lines 90-94: "A growing number of laser treatments facilitates" must be clarified. Also, provide further justification for why it is relevant to include the etiologies of the selected skin conditions.

Due to the word count of the introduction, we deleted this section of the introduction.

For peer review only - http://bmjopen.bmj.com/site/about/guidelines.xhtml

For peer review only

3. Line 96: Clarify what is meant by "diversity in laser devices." Are you referring to types of lasers? Settings? Etc.

We mean with 'diversity in laser devices' that there are many types of lasers. This is indicated on page 11, table 1, Inclusion criteria 'intervention' described as 'any type of laser treatment for vascular, pigmented or inflammatory lesions, tumours, scars, hair-related and (pre)malignant skin conditions'.

4. Line 106: Reference 11 refers to a paper that discusses intense pulsed light therapy as opposed to laser therapy for treating dermatologic conditions. Given that IPL differs from laser therapy, this reference must be corrected or the protocol must clearly state that the registry will be developed for laser therapy and IPL.

We agree with this statement of the reviewer. We removed this reference and corrected for another reference in which a great number of case reports in shown in the field of laser dermatology.

5. Line 107: Provide an example or reference to justify opinion that only successful studies are published.

We included an example of a reference to justify this statement : see references

Atakpo P, Vassar M. Publication bias in dermatology systematic reviews and meta-analyses. J Dermatol Sci. 2016;82:69–74, reference number 6.

6. Line 110/111: Provide references/justification for the mentioned PROs.

We provided references (7,8) for the justification of the mentioned PROs

7. Line 114/115: This statement is unclear as is, please re-word.

We removed the sentence.

8. Line 117: Please specify what the "evidence-based approach" is for…?

We removed the sentence and changed the sentence to 'To address the variations in outcome reporting, organizations such as the Core Outcome Measures in Effectiveness Trials Initiative (COMET) bring together researchers interested in developing a standardized set of core outcomes (COS) in various health-related fields. (Introduction, page 6, line 145)

9. Line 124: Please clarify which "comparisons" you are referring to and their significance.

We clarified in the following sentence which comparisons I am referring to and their significance:

Currently, 17 COS initiatives have been supported by CS-COUSIN in dermatology. These projects involve 26 different skin diseases, such as acne, atopic eczema, hidradenitis suppurativa, melanoma, nail psoriasis, rosacea, and vitiligo. However, with hundreds of different and mostly unrelated dermatoses that are treated with lasers in the field of laser dermatology, the need for a generic set of outcomes (GOS) is commanding. (introduction, page 6, line 157)

10. Line 129: Please clarify if "different laser treatments" refers to treatments with different types of lasers.

See scope page 8, sentence 188-190. The sentence has been changed to "The GOS is intended for use in the LEAD registry, with the focus on prospectively recording the effectiveness and safety of cutaneous non cosmetic laser interventions.".

11. Lines 168-173: Overall, the scope and applicability of outcomes is unclear. The authors should provide further justification for exclusions and must also clarify what is meant by "distinctive mode of action."

We adjusted the scope and added more detailed information, see page 8 line 185-193. The distincitive mode of actions refers to energy based devices with more than one wavelength and the role of drug delivery. Our focus is the effect of laser on skin conditions by means of one wavelength without the action of drug delivery or any other component.

12. Line 201- Table- Please clarify the inclusion criteria for the patient population. Also, provide further justification for the exclusion criteria for the intervention.

For peer review only - http://bmjopen.bmj.com/site/about/guidelines.xhtml

We changed the protocol and included patients with age 18 and older with vascular, pigmented or inflammatory lesions, benign tumours, scars, and hair follicle-related skin conditions treated by lasers. With these inclusion criteria we refer to all medical indications for which laser could be an effective treatment. With the excluision criteria 'Non-humans, flebological skin conditions, laser assisted drug delivery, low laser level therapy, body-contouring, skin tightening, hair removal, rejuvenation and anti-aging' we justify that we do not focus on cosmetic indications and energy based devices with more than one wavelength, additional drug delivery and focusing on cosmetic outcomes, see methods, table 1, page 11.

13. Lines 248-250: Please clarify if participants will be invited via mail or email. If an email is used, will the email provide a link to the questionnaires or will a subsequent email be provided? Also, please specify where information related to consenting to participate will be included.

Those who want to participate will be asked to respond with their name, country of origin and email address. We described now that 'Participants will be invited to participate in web-based anonymized electronic questionnaires. The surveys will be administered using Lime Survey and will be accessible via a direct hyperlink from the invitation email'.

14. Lines 259-266: Please provide further explanation for the potential outcomes listed.
We removed the alinea of potential outcomes.

15. Lines 274-275: The authors' state that participants will be recruited from Europe for "ensuring an international context" however, one could argue that this is misleading as participants from other parts of the world will not be included. Could consider re-wording to state "diverse context."
We reconsidered the European scope. A major change is that we have changed the scope registry from European to international scope. By sending the survey invitation to experts and advocacy groups from different continents, we aim to reflect a broad range of patients and health professionals with diverse backgrounds and experiences. Our steering committee now consists of laser experts from all continents to ensure the international context.

16. Lines 310-313: Please explain how the results will be "fed-back." Also, elaborate on who the included feedback will be from and how it will be presented (i.e. individual comments, summary of findings, etc.)
Results will be a feedback given in the form of charts. In these charts, summary of findings, votings, of each of the Delphi questions will be given to both groups of patients and health care professionals by email.

17. Line 331: Please specify who the target audience is for the international conference.
The target audience will be experts in the field of laser dermatology and physicians who work with lasers .

18. Line 334-367: Overall, a greater level of detail must be provided in the discussion section (i.e. provide examples of identified outcomes; specify what methodological guidelines you are referring to; clarify if "multiple treatments" means several treatments with the same device or treatments with different devices; clarify what is meant by "a diversity of outcome items;" elaborate on what you will gain from having a diverse sample of participants)
Examples of identified outcomes are given in the manuscript. We elaborated on strengths of methodological guidelines and the gain form having a divers sample of participant in the discussion section, see line 377-380, words "There are several strengths using the Delphi method for this study. First, the Delphi method allows to recruit a large number of laser experts, physicians and patients from diverse regions globally. The diversity in the experts' backgrounds and expertise ensures maximum impact of the results."

19. Line 379: Please specify how you will ensure that the email from each participant will be kept separate from the online survey.
Only the investigator has a code that relates to the email of the participant, to make sure that the participant receives a reminder when not completing the survey. However, the answers of the survey will relate to the code and not directly to the email.

For peer review only - http://bmjopen.bmj.com/site/about/guidelines.xhtml

20. Line 382: Please specify how consent will be obtained and if consent will be repeated for each round of the process.

The process of how consent will be obtained is described under the section of 'Definition of consensus', page 15, line 339-346. The definition of consensus is presented in Table 2. 'Consensus in' is defined as approval of the outcome by the vast majority (70 %) of all stakeholder groups that score 7, 8, or 9 with fewer than the minority (15 %) of panelists scoring 1–3. On the contrary, 'consensus out' is defined as 70% or more of all stakeholder groups scoring as 1 to 3 and less than 15% scoring as 7 to 9 [12] After three e-Delphi rounds, outcomes will be classified as 'consensus in' (consensus on the importance of the outcome), 'consensus out' (no consensus on the importance, or consensus on nonimportance) or 'no consensus' (consensus on the importance in only one or or no consensus.

<u>Reviewer: 4</u>
Reviewer Name: Daniel Schlessinger
Institution and Country: Northwestern University Feinberg School of Medicine

Please leave your comments for the authors below
This is a well-written protocol for an important topic - the generation of a Core Outcome Set. I have a few questions, however:
1) Page 9, Line 193-194: the authors state that their literature search will be of the MEDLINE and EMBASE databases, "between January 2013 and December 2017". Why does the literature search terminate in December 2017? Certainly this will miss a number of important articles published in laser research between 12/2017 and the present date. At the earliest, the literature search should terminal in December 2018.

We thank the reviewer for studying our paper and providing positive feedback and comments. The literature search terminates in December 2017 as our study on outcome reporting started in January 2018. We performed a literature review which was in January 2018 the most updated version. For more details we refer to the systematic review which has recently been published:

Fransen F, Tio D, Prinsen CA, Haedersdal M, Hedelund L, Laubach HJ, Marini L, Paasch U, Passeron T, Wolkerstorfer A. A Systematic Review of Outcome Reporting in Laser Treatments for Dermatological Diseases. J Eur Acad Dermatol Venereol. 2019 Aug 30. doi: 10.1111/jdv.15928

2) The majority of Core Outcome Sets have been developed for a specific medical condition - not a medical treatment. This makes sense, as the most important outcomes are generally condition-specific. The author's proposal is fundamentally different, however. Lasers are used for an extremely diverse set of conditions and I expect the resultant outcomes generated in their long list to be multifarious and in some cases unrelated. How can one COS encompass all laser-related outcomes? Wouldn't it be better to focus this (e.g., for lasers as used for vascular malformations or abnormalities)?

We would like to thank the suggestion of the reviewer and we appreciate the valid assessment. Regarding the comments on the manuscript, the reviewer has raised an important issue on the methodological approach. One COS could simply not encompass all laser-related outcomes indeed.
However, the current COSs development suggest that the outcomes are most likely very similar for specific different skin conditions and their different treatments (see CS-COUSIN projects such as Vascular Malformations, Atopic Eczema, Vitiligo and Acne. However, given the long duration and huge effort of the COS development and validation process, it is impossible to reach consensus on the COS for each skin condition apart. All the more as there are hundreds of uncommon dermatological conditions for which laser treatments have been documented and published. Due to overlapping outcomes and the long process of development of a COS for each disease, we propose a generic set of outcomes for various skin diseases treated by laser.
To clarify the aforesaid purpose of the development generic outcomes for a registry, the following changes to the text in the manuscript:

Introduction section, page 6: line 153 " Since 2015, the international, multidisciplinary working group, the Cochrane Skin Group- Core Outcome Set Initiative (CS-COUSIN) has been established" until line 164 "Therefore we focus on developing a generic set of outcomes (GOS) for the purpose of the LEAD registry. The GOS is intended to be applied to the assessment of various, unrelated skin diseases that are treated with different lasers."

For peer review only - http://bmjopen.bmj.com/site/about/guidelines.xhtml

SUPPLEMENTARY FILE 1

LEAD Registry : Steering Committee

Coordination team

Frederike Fransen (the Netherlands)

Albert Wolkerstorfer (the Netherlands)

Phyllis Spuls (the Netherlands)

In addition to the coordinaton team , the LEAD registry Steering Committee includes:

Murad Alam (US),Ashraf Badawi (Egypt), Pablo Boixeda (Spain), Iltefat Hamzavi (US), Merete Haedersdal (Denmark), Lene Hedelund (Denmark), Kristen Kelly (US), Taro Kono (Japan), Hans-Joachim Laubach (Switzerland), Woraphong Manuskiatti (Thailand), Leonardo Marini (Italy), Keyvan Nouri (US), Uwe Paasch (Germany), Thierry Passeron (France), Sanna Prinsen (The Netherlands), Ines Verner (Israel)

For peer review only

<u>**Author information list**</u>

Frederike Fransen (the Netherlands)

Amsterdam UMC, Department of Dermatology, 9, 1105 AZ, Amsterdam, the Netherlands.

f.fransen@amsterdamumc.nl

Albert Wolkerstorfer (the Netherlands)

Amsterdam UMC, Department of Dermatology, 9, 1105 AZ, Amsterdam, the Netherlands

a.wolkerstorfer@amsterdamumc.nl

Phyllis Spuls (the Netherlands)

Amsterdam UMC, Department of Dermatology, 9, 1105 AZ, Amsterdam, the Netherlands

ph.i.spuls@amsterdamumc.nl

Murad Alam (US)

Department of Dermatology, Feinberg School of Medicine, Northwestern University, Chicago, IL, USA. m-alam@northwestern.edu.

Department of Dermatology, Northwestern Memorial Hospital, Arkes Family Pavilion, 676 N Saint Clair Suite 1600, Chicago, IL, 60611, USA. m-alam@northwestern.edu

m-alam@northwestern.edu

Ashraf Badawi (Egypt)

Dermatology Unit, Department of Medical Applications of Lasers (MAL), National Institute of Laser Enhanced Sciences, Cairo University, Giza, Egypt

ashrafbadawi@hotmail.com

Pablo Boixeda (Spain)

Dermatology Department, Ramón y Cajal Hospital, Madrid, Spain

pboixeda@gmail.com

Iltefat Hamzavi (US)

Department of Dermatology, Henry Ford Hospital, Detroit, MI, USA

For peer review only

IHamzavi@hamzavi.com

Merete Haedersdal (Denmark)

Massachusetts General Hospital, Harvard Medical School Boston, USA

University of Copenhagen, Bispebjerg Hospital, Denmark

mhaedersdal@dadlnet.dk

Lene Hedelund (Denmark)

Department of Dermatology, Aarhus University Hospital, Denmark

lenehede@rm.dk

Kristen Kelly (US)

Beckman Laser Institute, University of California, Irvine, California, USA

kmkelly@uci.edu

Taro Kono (Japan)

Department of Plastic and Reconstructive Surgery, Tokai University School of Medicine, Isehara, Japan.

tkono@tokai-u.jp

Hans-Joachim Laubach (Switzerland)

Department of Dermatology and Venereology, Geneva University Hospitals (HUG), Switzerland.

hlaubach@esld.eu

Woraphong Manuskiatti (Thailand)

Faculty of Medicine Siriraj Hospital, Department of Dermatology , Mahidol University , Bangkok , Thailand

woraphong.man@mahidol.ac.th

For peer review only

Leonardo Marini (Italy)

SDC - The Skin Doctors' Center, Trieste, Italy

leonardo.marini@skindoctors.it

Keyvan Nouri (US)

Dermatology and Cutaneous Surgery, University of Miami School of Medicine, 1475 NW 12th Ave., Miami, FL, 33136, USA

KNouri@med.miami.edu

Uwe Paasch (Germany)

Department of Dermatology, Venereology and Allergy, University of Leipzig

uwe.paasch@hautclinicum.de

Thierry Passeron (France)

University of Côte d'Azur, University Hospital Nice, Department of Dermatology, Nice, France

University of Côte d'Azur, Centre Méditéranéen de Médecine Moléculaire (C3M), INSERM U1065, team 12, Nice, France

Thierry.Passeron@unice.fr

Sanna Prinsen (The Netherlands)

Department of Epidemiology and Biostatistics, Amsterdam Public Health research institute, Amsterdam UMC, Vrije Universiteit Amsterdam, The Netherlands.

c.prinsen@vumc.nl

Ines Verner (Israel)

Verner Clinic, Tel Aviv, Israel.

ines.verner@gmail.com

For peer review only

SUPPLEMENTARY FILE 2

The definitions for COS, outcome, outcome instruments and outcome parameters according to Prinsen *et al*. (2014). [1]

Definitions

Similar constructs are defined differently across several research groups such as COMET, OMERACT, and HOME. As there is currently no consensus on the definitions, we would like to explicitly state the definitions that are being used in the COMET Delphi study in order to avoid any possible misinterpretations.

Core outcome set (COS)

A COS is an agreed minimum set of outcomes that should be measured and reported in all clinical trials of a specific disease or trial population. A COS includes all relevant outcomes of a specific health condition within a specified setting (the OMERACT definition refers to 'core domain set' whereas the HOME definition refers to 'core outcome domains').

Generic core outcome set (GOS)

A GOS is an agreed minimum set of *generic* outcomes that should be measured and reported in all clinical trials of a specific disease or trial population. In this study, the GOS is intended to be applied for the assessment of various, unrelated skin diseases that are treated with different types of lasers.

Outcome and outcome domain.

Throughout this report, the definition of "outcome" refers to a single construct that can be measured as a standalone item (e.g. 'erythema'), while the term "outcome domain" or "domain" is an umbrella term for a group of associated outcomes (e.g. 'signs as assessed by physician').

Outcome measurement instrument

An outcome measurement instrument refers to how the outcome is being measured (the tool used to assess the outcome). An outcome measurement instrument can be a single question, a questionnaire, a performance-based test, a physical examination, a laboratory measurement, an imaging technique, and so forth (the HOME definition refers to 'outcome measure').

Reference

1 Prinsen CAC, Vohra S, Rose MR, *et al.* Core Outcome Measures in Effectiveness Trials (COMET) initiative: Protocol for an international Delphi study to achieve consensus on how to select outcome measurement instruments for outcomes included in a 'core outcome set'. *Trials* 2014;**15**. doi:10.1186/1745-6215-15-247

For peer review only

SUPPLEMENTARY FILE 3

Systematic review search strategies

Pubmed

1.“Skin” [Majr MeSH]

2.“cutaneous” [Majr MeSH]

3.“dermatology” [Majr MeSH]

4. “Skin Diseases”

5. 1 or 2 or 3 or 4

6.“laser” [Majr MeSH]

7.“alexandrite laser” [MeSH Terms]

8 “laser, pulsed dye” [MeSH Terms]

9. “er yag” [MeSH Terms]

10. “laser, nd yag” [MeSH Terms]

11. “laser, ruby” [MeSH Terms]

12. “laser, ysgg” [MeSH Terms]

13. “laser, argon” [MeSH Terms]

14. “laser, ktp” [MeSH Terms]

15. “laser, q switched” [MeSH Terms]

16. “laser, carbon dioxide” [MeSH Terms]

17. “laser, co2” [MeSH Terms]

18. “laser, diode” [MeSH Terms]

19. “thullium laser”

20. “fluoride laser”

21. “fractional laser”

22. “fractional CO2 laser”

23. “non-ablative fractional laser”

For peer review only

24.“Humans[Mesh]

25. "last 5 years"[PDat]

26. 6 or 7 or 8 or 9 or 10 or 11 or 12 or 13 or 14 or 15 or 16 or 17 or 18 or 19 or 20 or 21 or 22 or 23

26. 5 and 26

Embase:

1. #1, Skin.mp. or exp skin/

2. #2, cutaneous.mp.

3. #3, dermatology.mp. or exp dermatology/

4. #4, skin diseases.mp. or exp skin disease/

5. #5, laser.mp. or exp laser/

6. #6, laser treatment.mp.

7. #7, laser therapy.mp.

8. #8, skin laser therapy.mp.

9. #9, exp argon laser/ or exp frequency doubled neodymium YAG laser/ or exp thulium YAG laser/ or exp dye laser/ or exp gallium aluminum arsenide laser/ or exp neodymium laser/ or exp pulsed dye laser/ or exp carbon dioxide laser/ or exp excimer laser/ or exp YAG laser/ or exp alexandrite laser/ or exp argon fluoride laser/ or exp gas laser/ or exp laser surgery/ or exp erbium YAG laser/

10. #10, nd YAG laser.mp.

11. #11, non-ablative fractional laser.mp.

12. #12, CO2 laser.mp.

13. #13, fractional CO2 laser.mp.

14. #14, carbon dioxide laser.mp. or exp carbon dioxide laser/

15. #15, q switched laser.mp.

16. #16, nd YAG laser.mp.

17. #17, exp symptom assessment/ or exp symptom/ or symptoms.mp.

18. #18, outcome assessment.mp. or exp outcome assessment/

19. #19, treatment outcome.mp. or exp treatment outcome/

For peer review only

20. #20, exp treatment outcome/ or exp outcome assessment/ or outcome.mp.

21. #1 or #2 or #3 or #4

22. #5 or #6 or #7 or #8 or #9 or #10 or #11 or #12 or #13 or #14 or #15 or #16

23. #17 or # 18 or #19 or #20

24. #21 and #22

25. #23 and #24

26. 25 and 2013:2017.(sa_year).

27. 26 and "human" [Subjects]

For peer review only

SUPPLEMENTARY FILE 4

A list of invited patient support groups for the Delphi survey

**Name of Society**
Hidradenitis Patiëntenvereniging (NL)
Nevus Netwerk Nederland (NL)
Nevus Outreach (US)
Nevus Support (AU)
Neurofibromatose Vereniging Nederland (NL)
The Neuro Foundation (UK)
Neurofibromatose Ireland Association (IE)
Vereniging Wijnvlek Sturgeweber syndroom (NL)
Schweizerischen Nuerofibromatose Vereinigung (CH)
Interessengemeinschaf Sturge-Weber-Syndrom (DE)
Sturge Weber Foundation Great Britain (UK)
Sturge-Weber-Foundation (US)
Vitiligo patientenvereniging (NL)
National Vitiligo Foundation (US)

For peer review only

BMJ Open

A generic outcome set for the international registry on Laser TrEAtments in Dermatology (LEAD): a protocol for a Delphi study to achieve consensus on what to measure

Journal:	*BMJ Open*
Manuscript ID	bmjopen-2020-038145.R1
Article Type:	Protocol
Date Submitted by the Author:	07-Mar-2020
Complete List of Authors:	Fransen, Frederike; Amsterdam UMC , Dermatology<br>Spuls, Phyllis; Department of Dermatology, Amsterdam Public Health, Infection and Immunity, Amsterdam University Medical Center<br>Alam, Murad; Northwestern Medical Faculty Foundation,<br>Badawi, Ashraf; Dermatology Unit, Department of Medical Applications of Lasers (MAL), National Institute of Laser Enhanced Sciences, Cairo University<br>Boixeda, Pablo; Dermatology Department, Ramón y Cajal Hospital<br>Haedersdal, Merete; Copenhagen University Hospital Bispebjerg, Dermatology<br>Hamzavi, Iltefat; Department of Dermatology, Henry Ford Hospital, Detroit<br>Hedelund, Lene; Aarhus Universitetshospital, Dermatology<br>Kelly, Kristen ; Beckman Laser Institute, University of California<br>Kono, Tara; Department of Plastic and Reconstructive Surgery, Tokai University School of Medicine<br>Laubach, Hans Joachim; Hopitaux Universitaires de Geneve, Dermatology and Venereology<br>Manuskiatti, Woraphong; Faculty of Medicine Siriraj Hospital, Department of Dermatology , Mahidol University<br>Marini, Leonardo; SDC - The Skin Doctors' Center, Dermatology<br>Nouri , Keyvan ; Dermatology and Cutaneous Surgery, University of Miami School of Medicine, 1475 NW 12th Ave.,<br>Paasch, Uwe; University of Leipzig<br>Passeron, Thierry; Centre Hospitalier Universitaire de Nice, Dermatology<br>Prinsen, C; VU University Medical Center,, 19Department of Epidemiology and Biostatistics, Amsterdam Public Health research institute, Amsterdam UMC<br>Verner, Ines; Verner Clinic<br>Wolkerstorfer, Albert; Academic Medical Center (AMC), Dermatology
**Primary Subject Heading**:	Dermatology
Secondary Subject Heading:	Evidence based practice
Keywords:	Laser therapy < DERMATOLOGY, DERMATOLOGY, Surgical dermatology < DERMATOLOGY

For peer review only - http://bmjopen.bmj.com/site/about/guidelines.xhtml

I, the Submitting Author has the right to grant and does grant on behalf of all authors of the Work (as defined in the below author licence), an exclusive licence and/or a non-exclusive licence for contributions from authors who are: i) UK Crown employees; ii) where BMJ has agreed a CC-BY licence shall apply, and/or iii) in accordance with the terms applicable for US Federal Government officers or employees acting as part of their official duties; on a worldwide, perpetual, irrevocable, royalty-free basis to BMJ Publishing Group Ltd ("BMJ") its licensees and where the relevant Journal is co-owned by BMJ to the co-owners of the Journal, to publish the Work in this journal and any other BMJ products and to exploit all rights, as set out in our licence.

The Submitting Author accepts and understands that any supply made under these terms is made by BMJ to the Submitting Author unless you are acting as an employee on behalf of your employer or a postgraduate student of an affiliated institution which is paying any applicable article publishing charge ("APC") for Open Access articles. Where the Submitting Author wishes to make the Work available on an Open Access basis (and intends to pay the relevant APC), the terms of reuse of such Open Access shall be governed by a Creative Commons licence – details of these licences and which Creative Commons licence will apply to this Work are set out in our licence referred to above.

Other than as permitted in any relevant BMJ Author's Self Archiving Policies, I confirm this Work has not been accepted for publication elsewhere, is not being considered for publication elsewhere and does not duplicate material already published. I confirm all authors consent to publication of this Work and authorise the granting of this licence.

For peer review only

A generic outcome set for the international registry on Laser TrEAtments in Dermatology (LEAD): a protocol for a Delphi study to achieve consensus on *what* to measure

Frederike Fransen[1], Phyllis I. Spuls[1], Murad Alam[2,3], Ashraf Badawi[4], Pablo Boixeda[5], Merete Haedersdal[6,7], Iltefat Hamzavi[8], Lene Hedelund[9], Kristen M. Kelly[10], Taro Kono[11], Hans-Joachim Laubach[12], Woraphong Manuskiatti[13], Leonardo Marini[14], Keyvan Nouri[15], Uwe Paasch[16], Thierry Passeron[17,18], Cecilia A.C. Prinsen[19], Ines Verner[20], Albert Wolkerstorfer[1]

[1] Department of Dermatology, Amsterdam Public Health, Infection and Immunity, Amsterdam University Medical Center, Amsterdam, the Netherlands [2]Department of Dermatology, Feinberg School of Medicine, Northwestern University, Chicago, IL, USA. [3]Department of Dermatology, Northwestern Memorial Hospital, Arkes Family Pavilion, 676 N Saint Clair Suite 1600, Chicago, IL, 60611, USA. [4]Dermatology Unit, Department of Medical Applications of Lasers (MAL), National Institute of Laser Enhanced Sciences, Cairo University, Giza, Egypt. [5]Dermatology Department, Ramón y Cajal Hospital, Madrid, Spain. [6]Massachusetts General Hospital, Harvard Medical School Boston, USA. [7]University of Copenhagen, Bispebjerg Hospital, Denmark. [8]Department of Dermatology, Henry Ford Hospital, Detroit, MI, USA. [9]Department of Dermatology, Aarhus University Hospital, Denmark. [10]Beckman Laser Institute, University of California, Irvine, California, USA. [11]Department of Plastic and Reconstructive Surgery, Tokai University School of Medicine, Isehara, Japan. [12]Department of Dermatology and Venereology, Geneva University Hospitals (HUG), Switzerland. [13]Faculty of Medicine Siriraj Hospital, Department of Dermatology , Mahidol University , Bangkok , Thailand. [14]SDC - The Skin Doctors' Center, Trieste, Italy. [15]Dermatology and Cutaneous Surgery, University of Miami School of Medicine, 1475 NW 12th Ave., Miami, FL, 33136, USA. [16]Department of Dermatology, Venereology and Allergy, University of Leipzig. [17]University of Côte d'Azur, University Hospital Nice, Department of Dermatology, Nice, France. [18]University of Côte d'Azur, Centre Méditéranéen de Médecine Moléculaire (C3M), INSERM U1065, team 12, Nice, France. [19]Department of Epidemiology and Biostatistics, Amsterdam

For peer review only - http://bmjopen.bmj.com/site/about/guidelines.xhtml

Public Health research institute, Amsterdam UMC, The Netherlands. [20]*Verner Clinic, Tel Aviv, Israel.*

Corresponding author

Frederike Fransen

Department of Dermatology, Amsterdam UMC

Meibergdreef 9

1105 AZ Amsterdam

Phone: +31 20 56 67 792

E-mail: frederikefransen@gmail.com

For peer r

Abstract word count: 245

Main text word count: 3039

Table count: 2

Figure count: 1

Supplementary Files: 4

References: 25

Funding sources statement:

This manuscript presents independent research that has been supported by a research grant from the European Academy of Dermatology and Venereology (EADV Project proposal reference number 2017-035).

Competing interests

There are no competing interests for any author.

ABSTRACT

Introduction: While laser technology has expanded the armamentarium of treatment for various skin diseases during the past years, heterogeneity in study outcomes hampers comparability and appropriate evidence synthesis. Part of these issues can be addressed by developing a generic outcome set. Using the Delphi method, this study aims to seek consensus between key stakeholders on relevant generic outcomes (*what* to measure) for implementation in the international registry on Laser trEAtments in Dermatology (LEAD). The registry is focused on collecting research data on various laser treatments for skin disorders.

Methods and analysis: By reviewing the literature and involvement of key stakeholder groups

For peer review only - http://bmjopen.bmj.com/site/about/guidelines.xhtml

and adult patients in need or after laser surgery and health professionals, a preliminary list of outcomes will be generated and categorized into domains. Using these outcomes, an international three-round Delphi study will be performed to rate the importance of outcomes in the selection of a generic outcome set. Participants are allowed to provide new outcomes to the prelimary list for revisions during the first Delphi round. Finally, results will be discussed during a consensus meeting to agree on generic outcomes to be used in the LEAD Registry.

For peer review only

Ethics and Dissemination: An ethics approval was not applicable (W19_290 # 18.336). The study is registered with the CS-COUSIN (Cochrane Skin Core OUtcome Set INitiative) and the Core Outcome Measures in Effectiveness Trials (COMET) initiative. Procedures will be conducted according to the Declaration of Helsinki. The findings will be disseminated through peer-reviewed publications and conference presentations.

Keywords: Laser Therapy, Dermatology, Consensus study, Delphi study, Disease registry, Generic Outcome Set

For peer review only - http://bmjopen.bmj.com/site/about/guidelines.xhtml

ARTICLE SUMMARY

Strengths and limitations of this study

•This protocol outlines the first international consensus effort to develop a generic outcome set for use in the international LEAD laser registry.

• With advances in laser technology, considering outcomes of importance (*what* to measure) to patients and health professionals is crucial.

•A comprehensive systematic review will explore which outcomes are used and reported in existing studies on laser treatments.

• The Delphi procedure requires three survey rounds and involves a large group of stakeholders across various disciplines and geographical areas including patients, reflecting different viewpoints.

INRODUCTION

For peer review only - http://bmjopen.bmj.com/site/about/guidelines.xhtml

During the past decades, modifications in laser technology have further widened its scope and greatly expanded the cutaneous laser surgeon's armamentarium [1,2]. Today, there are many medical indications in dermatology, encompassing vascular, pigmented, inflammatory, metabolic or infectious lesions, benign tumours, scars, and hair follicle- related skin conditions that are regularly - and sometimes exclusively - treated with lasers [1–3]. Many of these disorders meet the criteria of an orphan disease.

The diversity in laser devices and the spectrum of medical indications pose unique research challenges for clinical decision-making in laser therapy. Because most laser physicians are not exposed to large numbers of patients receiving laser treatments for uncommon indications, knowledge on the most effective laser treatment, including safety and used regimen, is unclear. The current evidence for most of these specific skin conditions is sporadic at best, consisting mostly of case reports and case series and only a very small number of randomized controlled trials (RCTs) [4,5]. Moreover, most often only isolated successes are reported while cases that failed to respond are not published, leading to publication bias [6]. Another issue hampering evidence synthesis is heterogeneity of outcome definition, measurement and reporting in laser research. Patient-reported outcomes (PROs), such as 'patient experience of laser treatments' and 'health-related quality of life', are often not reported and together with selective outcome reporting in laser research, it is all a serious threat to comparative effectiveness research as it limits the ability to compare, contrast, and combine individual studies [7,8]. As a result, this hampers to draw meaningful conclusions and guidance to inform clinical decision-making [9,10].

To overcome this issue in the field of laser dermatology, the development of the International Laser Treatment (LEAD) Registry has been proposed to initiate collaborative data pooling of a

For peer review only

wide range of skin disorders. The development of a registry may be the key to the lack of solid evidence for laser treatments in dermatology, however, well-defined standardized and generic outcomes are required for its establishment.

To address the variations in outcome reporting, organizations such as the Core Outcome Measures in Effectiveness Trials Initiative (COMET) bring together researchers interested in developing a standardized set of core outcomes in various health-related fields [11]. A core outcome set (COS) is defined as an agreed minimum set of outcomes that should be measured and reported in all clinical trials for a specific health condition, including methods used to measure these core outcomes[10,12]. Throughout this report, the definition of “outcome” refers to a single construct that can be measured as a standalone item (e.g. ‘erythema’), while the term “outcome domain” or “domain” is an umbrella term for a group of associated outcomes (e.g. ‘signs as assessed by physician’). Furthermore, the outcome instrument refers to how the outcomes are measured. Although a COS is recommended for clinical trials, they can also be developed for routine clinical practice, and for registries [10,12]. In 2015, the international, multidisciplinary working group, the Cochrane Skin Group- Core OUtcome Set INitiative (CS-COUSIN) has been established [13]. The organization supports dermatology-specific initiatives to develop and implement a COS by building upon experiences of the Harmonizing Outcome Measures for Eczema (HOME) initiative, which developed a roadmap to guide the process of COS development and implementation [14]. Currently, 17 COS initiatives have been supported by CS-COUSIN in dermatology. These projects involve 26 different skin diseases, such as acne, atopic eczema, hidradenitis suppurativa, melanoma, nail psoriasis, rosacea, and vitiligo [11,15]. However, with hundreds of different and mostly unrelated dermatoses that are treated with lasers in the field of laser dermatology, the need for a generic

For peer review only

outcome set (GOS) is commanding. Therefore we focus on developing a GOS (*what* to measure) for the purpose of the LEAD registry. The GOS is intended to be applied for the assessment of various, unrelated skin diseases that are treated with different types of lasers.

In summary, there is an urgency of using the same generic outcomes in laser therapy. Hence, establishing consensus on the relevant outcomes for the LEAD registry will promote clinical researchers to use outcomes chosen by consensus that are relevant to patients and clinicians. The use of generic outcomes support data synthesis for many diseases in dermatology. The protocol outlines the context, scope and methods for the development of a GOS to be implemented in the LEAD registry.

Aims and objectives

Aim

The aim of this study is to reach consensus between various stakeholders on generic outcomes relevant for the LEAD registry.

Objectives

Our study objectives are:

1. To identify outcomes that have previously been used and reported in RCTs, cohort studies, case-control studies and case series from a literature review and classify these outcomes into domains according to the COMET taxonomy;

For peer review only - http://bmjopen.bmj.com/site/about/guidelines.xhtml

2. To reach consensus between stakeholders on the outcomes of a GOS to be implemented in the LEAD registry.

Scope and applicability of outcomes

The registry is envisioned to suit all types of laser interventions for skin disorders in dermatology including vascular, pigmented or inflammatory lesions , benign tumours, scars, and hair follicle-related skin conditions treated with lasers. The GOS is intended for use in the LEAD registry, with the focus on prospectively recording the effectiveness and safety of cutaneous non cosmetic laser interventions. Therefore we excluded laser assisted drug delivery, low laser level therapy, body- contouring, skin tightening, hair removal, rejuvenation and anti-aging procedures. Furthermore, because of the distinctive mode of action and use in daily clinical practice, laser assisted drug delivery, low laser level therapy and laser procedures for (leg) veins were excluded.

For peer review only

METHODS AND ANALYSIS

Research group

The steering committee (FF, PS, AW, MA, AB, PB, IH, MH, LH ,KK, TK, HL, WM, LM, KN, UP, TP, CP, IV) provide input at critical points of the study such as protocol development, stakeholder recruitment, consensus process and the consensus meeting. Three members of the steering committee (FF,PS,AW) coordinate the overall project, ensure methodological quality of the project and make key decisions. All members of the steering committee will participate in the Delphi procedure as well as in the final consensus meeting. The steering committee has

For peer review only - http://bmjopen.bmj.com/site/about/guidelines.xhtml

representatives from The Netherlands, Denmark, Egypt, France, Germany, Israel, Italy, Japan, Spain, Switzerland, Thailand and USA, with extensive expertise in various laser treatments, outcomes research and clinical research. A list of all members of the steering committee is given in supplementary file 1.

Study design

Figure 1 provides a brief overview of the stepwise approach with different research methods. The study consists of the following two phases:

Phase 1: Identification of potential outcomes important in laser treatments by means of a

1. A systematic review to form the preliminary list of outcomes for the Delphi survey

2. Classification of outcomes into domains according to the COMET taxonomy

Phase 2: A consensus process involving key stakeholders who are able to suggest additional outcomes during the first round and who will rate the importance of outcome for reaching consensus on the GOS by means of a

1. Three-round Delphi survey.

2. Expert consensus meeting. attended by representatives of all stakeholder groups.

For peer review only

For peer review only - http://bmjopen.bmj.com/site/about/guidelines.xhtml

This study is registered with the CS-COUSIN and COMET initiative [11,16]. Results of the consensus study will be reported according to the Core Outcome Set-STAndards for Reporting (COS-STAR) [17].

Phase 1: Identification of potential outcomes and domains

Phase 1.1: Systematic literature review

The first phase of the study is to identify which outcomes should be measured and reported in a registry on laser treatments for skin disorders (what to measure: the GOS, see definitions in supplementary file 2). A SR will be performed to explore existing outcomes that are used in laser studies. According to the COMET guidelines [18], searches will be performed in the following database: MEDLINE and EMBASE. Articles between January 2013 and December 2017 will be retrieved. The electronic search strategy is detailed in supplementary file 3. A recent 5-year time period has been selected for the search so that outcomes extracted represent the practice of present-day laser research. The inclusion and exclusion criteria are presented in Table 1. Two reviewers will select articles and extract the data independently. Disagreement will be resolved by discussion and by consulting a third review author if necessary. The following data will be extracted from the selected articles in data extraction tables : authors, years of publication, country, cutaneous indications for treatment and type of laser treatments. We will assess what outcomes and outcome measurement instrument are used, consistency in outcomes, number of times an outcome was used, consistency in classification used.

For peer review only - http://bmjopen.bmj.com/site/about/guidelines.xhtml

Table 1 Inclusion and exclusion criteria for literature review

	Inclusion criteria	Exclusion criteria
Patient population and indication	Studies including patients age 18 and older with vascular, pigmented, inflammatory, metabolic or infectious lesions, benign tumours and hair follicle-related skin conditions treated with lasers	Non-humans flebological skin conditions Laser assisted drug delivery, low laser level therapy, body-contouring, skin tightening, hair removal, rejuvenation and anti-aging
Study design	RCTs, cohort studies, case-control studies, case series	In vitro studies, systematic reviews, abstracts and expert opinions, case reports
Intervention	Any type of laser treatment for vascular, pigmented or inflammatory lesions, benign tumours, and hair follicle-related skin conditions.	Laser assisted drug delivery, low laser level therapy, laser therapy for leg veins and cosmetic interventions (see scope of outcomes)
Outcomes		Non-clinical outcomes e.g. biochemical outcomes, imaging, confocal laser, histology
Publication	All studies are conducted between 2013-2017	

For peer review only - http://bmjopen.bmj.com/site/about/guidelines.xhtml

Phase 1.2: Classification of outcomes into domains

Subsequently, data will be classified according to the standardized taxonomy for outcomes proposed by the COMET initiative [19]. This taxonomy encompasses 38 domains within 5 core areas: mortality/survival; physiological/clinical; life impact; resource use; adverse events.

Outcomes and their classification in domains will be discussed with three members (FF, PS, AW) of the steering committee. The preliminary list of outcomes classified to domains will be included in the consensus process.

Phase 2: Consensus process

Phase 2.1: Delphi procedure

For investigating crucial outcomes in context of the LEAD registry, a Delphi study will be conducted. The Delphi is based on a structured process for gathering and condensing knowledge from key stakeholder groups by means of 3 rounds with a series of questionnaires [20]. The procedure will consist of three online rounds (Figure 1).

For peer review only - http://bmjopen.bmj.com/site/about/guidelines.xhtml

Participants

The involvement of a variety of stakeholders is a key part for the identification of outcomes and strongly recommended by methodologists [21].

The following representatives from four international key stakeholder groups are involved in the process of reaching consensus on outcomes:

1. Patients of age 18 with vascular, pigmented, inflammatory, metabolic or infectious lesions, benign tumours and hair follicle-related skin conditions treated by lasers.

2. Patient representatives involved in patient associations that raise awareness on the impact of vascular, pigmented, inflammatory, metabolic or infectious lesions, benign tumours and hair follicle-related skin conditions.

3. Health care professionals – Laser experts who treat patients with vascular, pigmented or inflammatory, metabolic or infectious lesions, benign tumours, hair follicle-related skin conditions and who are involved in research on laser treatments.

4. Health care professionals –General physicians who treat patients with dermatological indications.

Panel size and recruitment

There is no robust guidance for calculating the number of participants needed for a Delphi study and expectations are based on COMET Initiative guidelines and previous literature [16,22,23]. As there are various stakeholder groups involved in the Delphi procedure, we will recruit as many

For peer review only - http://bmjopen.bmj.com/site/about/guidelines.xhtml

international representatives as possible from each group. All potential participants will be invited with a letter explaining the aims and details of the study and the rationale and importance of completing the entire Delphi process. Respondents who agree to take part will be assigned a unique identification number. Furthermore, each member of the steering committee will be asked to cascade the link of the survey to 3 other physicians in their network. Patients and patient representatives will be recruited from national and international support groups for skin diseases treated with lasers and can be found in supplemental file 4. In addition, laser experts from the steering committee will be asked to recruit 3 patients with different skin conditions treated with lasers in their center. To make sure that we involve skin diseases of different categories, laser experts will indicate the diagnosis of the patients that are recruited. By sending the survey invitation to experts and patient support groups from different continents, we aim to reflect a broad range of patients and health professionals with diverse backgrounds and experiences. For each round, the number of participants invited and those who completed the surveys will be documented. The participants will have 3 weeks to complete each round. We will send personal reminder emails to those who did not respond after 7 and 14 days to increase the response rate.

Delphi survey

Participants will be divided into a group of patient and a group of health professional , leading to separate scoring of outcomes. All participants will be asked to rate the importance of each of the outcomes using the GRADE (Grading of Recommendations Assessment, Development and Evaluations) approach. The scale will range from 1 to 9 and will be categorized as follows: 1–3

For peer review only - http://bmjopen.bmj.com/site/about/guidelines.xhtml

'not important'; 4–6 'important but not critical'; and 7–9 'critical' [24,25]. If participants feel unable to rate or provide feedback they can select 'unable to score'.

Delphi rounds

Delphi round 1 .

During the first round of the Delphi survey, baseline characteristics (age, gender, country of practice) will be obtained from all participants. Patients will be asked for their medical indication and type of laser treatment, and whether any complications have occurred during treatment. Health professionals will be asked their specialty (laser dermatology, general dermatology or other), workplace (academic, teaching hospital or non-teaching hospital) and years in practice. Next, participants will be asked to score listed outcomes and will have the option to suggest any additional outcomes that are not yet presented in the preliminary list.

Delphi round 2 and 3

In the second and third Delphi rounds, all participants will receive feedback on the scores of the previous round in both the patient and the health professional group. The outcomes from the previous rounds will be presented with the median scores from each stakeholder group combined with a histogram showing the scoring distribution. Subsequently, participants will be asked to score all outcomes for which consensus has not been reached, in the same manner as in the first Delphi round. Outcomes for which there was only consensus within a single stakeholder group will also be shown to the other stakeholder group to evaluate whether consensus can be achieved in both stakeholder groups.

For peer review only - http://bmjopen.bmj.com/site/about/guidelines.xhtml

Definition of consensus

The definition of consensus is presented in Table 2. 'Consensus in' is defined as approval of the outcome by the vast majority (70 %) of all stakeholder groups that score 7, 8, or 9 with fewer than the minority (15 %) of panelists scoring 1–3. On the contrary, 'consensus out' is defined as 70% or more of all stakeholder groups scoring as 1 to 3 and less than 15% scoring as 7 to 9 [12]. After three e-Delphi rounds, outcomes will be classified as 'consensus in' (consensus on the importance of the outcome), 'consensus out' (no consensus on the importance, or consensus on nonimportance) or 'no consensus' (consensus on the importance in only one or or no consensus).

Table 2: Definitions of consensus for identifying generic outcomes for the LEAD registry

Consensus category	Clarification	Definition
Consensus in	Outcome should be included in the registry	70% of stakeholder groups scoring as 7 to 9 and < 15% of stakeholder groups scoring as 1 to 3
Consensus out	Outcome should not be included in the registry	70% or more of stakeholder groups scoring as 1 to 3 and < 15% of stakeholder groups scoring as 7 to 9
No consensus	Hesitation about relevance of outcome to be included in the registry	Anything other

Phase 2.2: Determination of the GOS during the expert consensus meeting

In case complete consensus is reached in the Delphi procedure on the outcomes of the GOS , no formal consensus meeting will be organized. However, the results of the Delphi will be discussed with three members of the steering committee (FF. PS, AW) to check misconceptions in the Delphi method and to safeguard a well-defined GOS. For outcomes for which consensus

For peer review only - http://bmjopen.bmj.com/site/about/guidelines.xhtml

definition during the Delphi has not been reached, we invite 15 participants from across all stakeholder groups to participate in an online expert consensus meeting within 2 months after the close of round 3. The primary goal of the meeting is discussing the 'no consensus' outcomes. Consensus results from the Delphi can be reversed in this meeting if reasons are very strong and clear.

Patient and public involvement

Patient and public were not involved in the development of this study protocol. However, patients will be involved and included within the Delphi procedure as expert group. Consensus methodology will ensure that the opinions and preferences of patients will be given the same weighting as those of the laser experts and health professionals. Furthermore, patients will participate in the final consensus meeting. We disseminate the main results to study participants and patients by email which will include a copy of the final outcomes of the GOS. In addition, where approval has been given, participants (including members of the public) will be named as contributors in the acknowledgments section.

DISCUSSION

By the end of this study, we hope to reach consensus on a GOS that could be implemented in an international registry with a research focus, that collects data of rare skin diseases treated by

For peer review only - http://bmjopen.bmj.com/site/about/guidelines.xhtml

lasers. Analysis of registry data provides insight into effectiveness and safety of different laser treatments across many skin diseases, laser centers and countries.

There are several strengths using the Delphi method for this study. First, the Delphi method allows to recruit a large number of laser experts, physicians and patients from diverse regions globally. The diversity in the experts' backgrounds and expertise ensures maximum impact of the results. Secondly, the Delphi method is the accurate tool in consensus processes in various stakeholder groups as individuals are able to express their own opinions and feedback can be provided in a controlled anonymous way. This means that there is room for individual disagreement but also consideration of the answers given by other individuals and stakeholder groups as a whole. However, there are also limitations of the Delphi method. Results are dependent upon the composition of the participants. There is a risk of relative uneven representations among patients, but also health professionals. Especially, when focusing on a specific group of rare skin diseases, selection bias could result in insufficient representation of other skin disorders. We request health professionals of the steering committee to recruit patients with 3 different skin disorders. Through this method, we hope to ensure that all subgroups including vascular, pigmented, metabolic, inflammatory lesions, benign tumours and hair follicle-related skin conditions, will be adequately involved. For patients it might be a barrier to imagine what is important to be included in a registry for a broad range of diseases, rather than one disease that is important to themselves. We will stress the importance of agreeing on

For peer review only - http://bmjopen.bmj.com/site/about/guidelines.xhtml

a GOS for all diseases in each round of the Delphi survey and consensus meetings. Photographs will be included to illustrate the variety of skin disorders that are involved. To provide the highest possible input we will extend our invitation to take part in the Delphi survey to patients and health professionals in Africa, Asia, South-America, Australia, in addition to Europe and North-America. With support from all panel members we hope to ensure that the LEAD registry will be internationally relevant, accepted and ready to use.

Trial status

The identification of generic outcomes for registry use is ongoing and in the initial phase. A systematic review has been performed to explore current outcomes used and reported in laser dermatology. We are currently preparing to recruit participants for the Delphi study. The generic outcomes s are expected to be implemented in the laser registry in 2020.

ETHICS AND DISSEMINATION

The medical research ethics committee of the Academic Medical Center Amsterdam confirmed that the Dutch Medical Research Involving Human Subjects Act does not apply to this study (W19_290 # 18.336) and that complete approval of this study by the committee is not necessary. All participants involved in the Delphi study will be asked for their consent before taking part. All procedures will be conducted according to the Declaration of Helsinki. All results from the consensus study will be reported in peer-reviewed indexed journals. The data will be presented at conferences chosen to reach a wide range of knowledge users.

For peer review only - http://bmjopen.bmj.com/site/about/guidelines.xhtml

For peer review only

Abbreviations

COMET: Core Outcome Measures for Effectiveness Trials; GOS: Generic Outcome Set; CSG-COUSIN: Cochrane Skin Group—Core Outcome Set Initiative; COSMIN: COnsensus-based Standards for the selection of health Measurement Instruments; GOS: Generic Outcome Set; GRADE: Grading of Recommendations Assessment; LEAD registry: Laser TrEAtment Dermatology registry; RCT: Randomized controlled trial.

For peer review only

Contributors

FF initiated the protocol, designed the study, wrote the manuscript and reviewed it for important intellectual content. PS contributed significantly to the study design and reviewed the manuscript for important intellectual content. CP contributed to the study design and reviewed the manuscript for important intellectual content. AW initiated the protocol, designed the study and reviewed it for important intellectual content. All authors (FF, PS, AW, MA, AB, PB, IH, MH, LH ,KK, TK, HL, WM, LM, KN, UP, TP, CP, IV) read and approved the final manuscript.

Acknowledgements

We are grateful to Jan Kottner of the CS-COUSIN methods group for providing advice for methodological issues during the protocol development. We acknowledge Marjolein

For peer review only - http://bmjopen.bmj.com/site/about/guidelines.xhtml

van Kessel as patient advocate of Naevus International for her support in preparing the Delphi rounds.

Supplementary Files

1. List of members of the LEAD registry steering committee
2. A glossary on the definitions of terminology
3. Search strategy for the systematic review of literature
4. A list of invited patient support groups for the Delphi survey

REFERENCES

1 Tanzi EL, Lupton JR, Alster TS. Lasers in dermatology: Four decades of progress. J. Am. Acad. Dermatol. 2003;**49**:1–31. doi:10.1067/mjd.2003.582

2 Husain Z, Alster TS. The role of lasers and intense pulsed light technology in dermatology. Clin. Cosmet. Investig. Dermatol. 2016;**9**:29–40. doi:10.2147/CCID.S69106

3 Houk LD, Humphreys T. Masers to magic bullets: an updated history of lasers in dermatology. *Clin Dermatol* 2007;**25**:434–42. doi:10.1016/j.clindermatol.2007.05.004

4 U.S. National Institutes of Health. ClinicalTrials.gov. clinicaltrials.gov. 2013.http://clinicaltrials.gov/ (accessed 28 Mar 2018).

5 Fransen F, Tio D, Prinsen CAC, *et al.* A Systematic Review of Outcome Reporting in Laser Treatments for Dermatological Diseases. *J Eur Acad Dermatology Venereol* Published Online First: 2019. doi:10.1111/jdv.15928

6 Atakpo P, Vassar M. Publication bias in dermatology systematic reviews and meta-analyses. *J Dermatol Sci* Published Online First: 2016. doi:10.1016/j.jdermsci.2016.02.005

7 Snyder CF, Aaronson NK, Choucair AK, *et al.* Implementing patient-reported outcomes assessment in clinical practice: A review of the options and considerations. Qual. Life Res. 2012. doi:10.1007/s11136-011-0054-x

8 Prinsen CAC, Mokkink LB, Bouter LM, *et al.* COSMIN guideline for systematic reviews of patient-reported outcome measures. *Qual Life Res* Published Online First: 2018. doi:10.1007/s11136-018-1798-3

9 Kirkham JJ, Gorst S, Altman DG, *et al.* COS-STAR: A reporting guideline for studies developing core outcome sets (protocol). *Trials* 2015;**16**. doi:10.1186/s13063-015-0913-9

10 Clarke M. Standardising outcomes for clinical trials and systematic reviews. Trials. 2007;**8**. doi:10.1186/1745-6215-8-39

11 http://www.comet-initiative.org/

12 Williamson PR, Altman DG, Blazeby JM, *et al.* Developing core outcome sets for clinical trials: Issues to consider. *Trials* Published Online First: 2012. doi:10.1186/1745-6215-13-

For peer review only - http://bmjopen.bmj.com/site/about/guidelines.xhtml

132

13 Schmitt J, Deckert S, Alam M, *et al.* Report from the kick-off meeting of the Cochrane Skin Group Core Outcome Set Initiative (CSG-COUSIN). In: *British Journal of Dermatology*. 2016. 287–95. doi:10.1111/bjd.14337

14 Schmitt J, Apfelbacher C, Spuls PI, *et al.* The Harmonizing Outcome Measures for Eczema (HOME) Roadmap: A Methodological Framework to Develop Core Sets of Outcome Measurements in Dermatology. *J Invest Dermatol* Published Online First: 2015. doi:10.1038/jid.2014.320

15 Prinsen CAC, Spuls PI, Kottner J, *et al.* Navigating the landscape of core outcome set development in dermatology. *J Am Acad Dermatol* Published Online First: 2019. doi:10.1016/j.jaad.2019.03.009

16 Williamson P. The comet initiative. *Trials* 2013;**0**:68DUMMY.http://ovidsp.ovid.com/ovidweb.cgi?T=JS&CSC=Y&NEWS=N&PAGE=fulltext&D=emed13&AN=71799862%5Cnhttp://oxfordsfx.hosted.exlibrisgroup.com/oxford?sid=OVID:embase&id=pmid:&id=doi:&issn=1745-6215&isbn=&volume=14&issue=&spage=68DUMMY&pages=68DUMMY&date=2013&title=

17 Kirkham JJ, Gorst S, Altman DG, *et al.* Core Outcome Set–STAndards for Reporting: The COS-STAR Statement. *PLoS Med* 2016;**13**. doi:10.1371/journal.pmed.1002148

18 Prinsen CAC, Vohra S, Rose MR, *et al.* Core Outcome Measures in Effectiveness Trials (COMET) initiative: Protocol for an international Delphi study to achieve consensus on how to select outcome measurement instruments for outcomes included in a 'core outcome set'. *Trials* 2014;**15**. doi:10.1186/1745-6215-15-247

19 Dodd S, Clarke M, Becker L, *et al.* A taxonomy has been developed for outcomes in medical research to help improve knowledge discovery. *J Clin Epidemiol* 2018;**96**:84–92. doi:10.1016/j.jclinepi.2017.12.020

20 Jones J, Hunter D. Consensus methods for medical and health services research. *Br Med J* Published Online First: 1995. doi:10.2164/jandrol.111.015065

21 Harman NL, Bruce IA, Kirkham JJ, *et al.* The importance of integration of stakeholder views in core outcome set development: Otitis Media with Effusion in children with cleft palate. *PLoS One* Published Online First: 2015. doi:10.1371/journal.pone.0129514

22 Young A, Brookes S, Rumsey N, *et al.* Agreement on what to measure in randomised controlled trials in burn care: Study protocol for the development of a core outcome set. *BMJ Open* 2017;**7**. doi:10.1136/bmjopen-2017-017267

23 Schlessinger DI, Iyengar S, Yanes AF, *et al.* Development of a core outcome set for clinical trials in squamous cell carcinoma: Study protocol for a systematic review of the literature and identification of a core outcome set using a Delphi survey. *Trials* 2017;**18**. doi:10.1186/s13063-017-2069-2

24 Balshem H, Helfand M, Schünemann HJ, *et al.* GRADE guidelines: 3. Rating the quality of evidence. *J Clin Epidemiol* Published Online First: 2011. doi:10.1016/j.jclinepi.2010.07.015

25 The Grading of Recommendations Assessment Development and Evaluation. GRADE working group. *Grade* 2014.

For peer review only

For peer review only - http://bmjopen.bmj.com/site/about/guidelines.xhtml

Figure legends

Figure 1: Flow diagram outlining the development of a generic outcome set for the LEAD registry.
Preparatory stages and process of consensus for relevant generic outcomes are summarized.

For peer review only

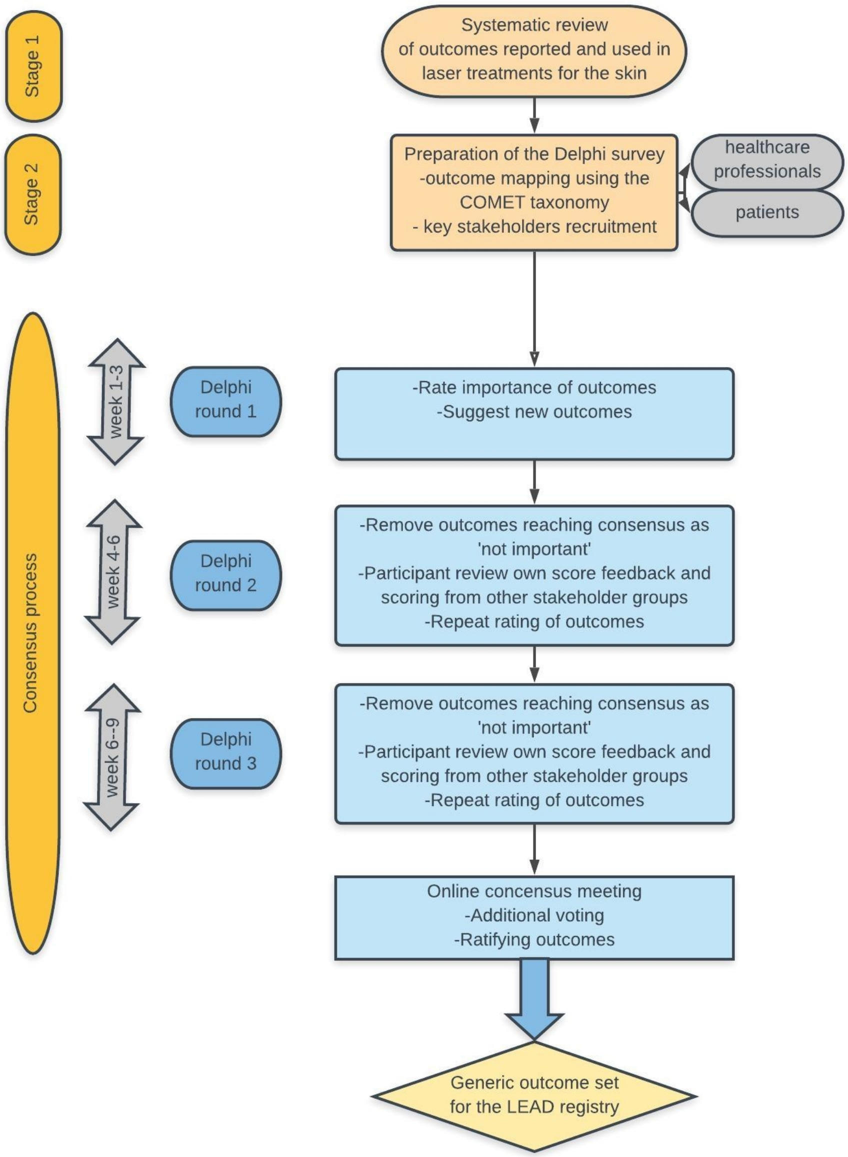

Flow diagram outlining the development of a generic outcome set for the LEAD registry.

206x277mm (160 x 160 DPI)

SUPPLEMENTARY FILE 1

LEAD Registry : Steering Committee

<u>*Coordination team*</u>

Frederike Fransen (the Netherlands)

Albert Wolkerstorfer (the Netherlands)

Phyllis Spuls (the Netherlands)

In addition to the coordinaton team , the LEAD registry Steering Committee includes:

Murad Alam (US),Ashraf Badawi (Egypt), Pablo Boixeda (Spain), Iltefat Hamzavi (US), Merete Haedersdal (Denmark), Lene Hedelund (Denmark), Kristen Kelly (US), Taro Kono (Japan), Hans-Joachim Laubach (Switzerland), Woraphong Manuskiatti (Thailand), Leonardo Marini (Italy), Keyvan Nouri (US), Uwe Paasch (Germany), Thierry Passeron (France), Sanna Prinsen (The Netherlands), Ines Verner (Israel)

For peer review only

Author information list

Frederike Fransen (the Netherlands)

Amsterdam UMC, Department of Dermatology, 9, 1105 AZ, Amsterdam, the Netherlands.

f.fransen@amsterdamumc.nl

Albert Wolkerstorfer (the Netherlands)

Amsterdam UMC, Department of Dermatology, 9, 1105 AZ, Amsterdam, the Netherlands

a.wolkerstorfer@amsterdamumc.nl

Phyllis Spuls (the Netherlands)

Amsterdam UMC, Department of Dermatology, 9, 1105 AZ, Amsterdam, the Netherlands

ph.i.spuls@amsterdamumc.nl

Murad Alam (US)

Department of Dermatology, Feinberg School of Medicine, Northwestern University, Chicago, IL, USA. m-alam@northwestern.edu.

Department of Dermatology, Northwestern Memorial Hospital, Arkes Family Pavilion, 676 N Saint Clair Suite 1600, Chicago, IL, 60611, USA. m-alam@northwestern.edu

m-alam@northwestern.edu

Ashraf Badawi (Egypt)

Dermatology Unit, Department of Medical Applications of Lasers (MAL), National Institute of Laser Enhanced Sciences, Cairo University, Giza, Egypt

ashrafbadawi@hotmail.com

Pablo Boixeda (Spain)

Dermatology Department, Ramón y Cajal Hospital, Madrid, Spain

pboixeda@gmail.com

Iltefat Hamzavi (US)

Department of Dermatology, Henry Ford Hospital, Detroit, MI, USA

For peer review only

IHamzavi@hamzavi.com

Merete Haedersdal (Denmark)

Massachusetts General Hospital, Harvard Medical School Boston, USA

University of Copenhagen, Bispebjerg Hospital, Denmark

mhaedersdal@dadlnet.dk

Lene Hedelund (Denmark)

Department of Dermatology, Aarhus University Hospital, Denmark

lenehede@rm.dk

Kristen Kelly (US)

Beckman Laser Institute, University of California, Irvine, California, USA

kmkelly@uci.edu

Taro Kono (Japan)

Department of Plastic and Reconstructive Surgery, Tokai University School of Medicine, Isehara, Japan.

tkono@tokai-u.jp

Hans-Joachim Laubach (Switzerland)

Department of Dermatology and Venereology, Geneva University Hospitals (HUG), Switzerland.

hlaubach@esld.eu

Woraphong Manuskiatti (Thailand)

Faculty of Medicine Siriraj Hospital, Department of Dermatology , Mahidol University , Bangkok , Thailand

woraphong.man@mahidol.ac.th

For peer review only

Leonardo Marini (Italy)

SDC - The Skin Doctors' Center, Trieste, Italy

leonardo.marini@skindoctors.it

Keyvan Nouri (US)

Dermatology and Cutaneous Surgery, University of Miami School of Medicine, 1475 NW 12th Ave., Miami, FL, 33136, USA

KNouri@med.miami.edu

Uwe Paasch (Germany)

Department of Dermatology, Venereology and Allergy, University of Leipzig

uwe.paasch@hautclinicum.de

Thierry Passeron (France)

University of Côte d'Azur, University Hospital Nice, Department of Dermatology, Nice, France

University of Côte d'Azur, Centre Méditéranéen de Médecine Moléculaire (C3M), INSERM U1065, team 12, Nice, France

Thierry.Passeron@unice.fr

Sanna Prinsen (The Netherlands)

Department of Epidemiology and Biostatistics, Amsterdam Public Health research institute, Amsterdam UMC, Vrije Universiteit Amsterdam, The Netherlands.

c.prinsen@vumc.nl

Ines Verner (Israel)

Verner Clinic, Tel Aviv, Israel.

ines.verner@gmail.com

For peer review only

SUPPLEMENTARY FILE 2

The definitions for COS, outcome, outcome instruments and outcome parameters according to Prinsen *et al*. (2014). [1]

Definitions

Similar constructs are defined differently across several research groups such as COMET, OMERACT, and HOME. As there is currently no consensus on the definitions, we would like to explicitly state the definitions that are being used in the COMET Delphi study in order to avoid any possible misinterpretations.

Core outcome set (COS)

A COS is an agreed minimum set of outcomes that should be measured and reported in all clinical trials of a specific disease or trial population. A COS includes all relevant outcomes of a specific health condition within a specified setting (the OMERACT definition refers to 'core domain set' whereas the HOME definition refers to 'core outcome domains').

Generic core outcome set (GOS)

A GOS is an agreed minimum set of *generic* outcomes that should be measured and reported in all clinical trials of a specific disease or trial population. In this study, the GOS is intended to be applied for the assessment of various, unrelated skin diseases that are treated with different types of lasers.

Outcome and outcome domain.

Throughout this report, the definition of "outcome" refers to a single construct that can be measured as a standalone item (e.g. 'erythema'), while the term "outcome domain" or "domain" is an umbrella term for a group of associated outcomes (e.g. 'signs as assessed by physician').

Outcome measurement instrument

An outcome measurement instrument refers to how the outcome is being measured (the tool used to assess the outcome). An outcome measurement instrument can be a single question, a questionnaire, a performance-based test, a physical examination, a laboratory measurement, an imaging technique, and so forth (the HOME definition refers to 'outcome measure').

Reference

1 Prinsen CAC, Vohra S, Rose MR, *et al.* Core Outcome Measures in Effectiveness Trials (COMET) initiative: Protocol for an international Delphi study to achieve consensus on how to select outcome measurement instruments for outcomes included in a 'core outcome set'. *Trials* 2014;**15**. doi:10.1186/1745-6215-15-247

For peer review only

SUPPLEMENTARY FILE 3

Systematic review search strategies

Pubmed

1.“Skin” [Majr MeSH]

2.“cutaneous” [Majr MeSH]

3.“dermatology” [Majr MeSH]

4. “Skin Diseases”

5. 1 or 2 or 3 or 4

6.“laser” [Majr MeSH]

7.“alexandrite laser” [MeSH Terms]

8 “laser, pulsed dye” [MeSH Terms]

9. “er yag” [MeSH Terms]

10. “laser, nd yag” [MeSH Terms]

11. “laser, ruby” [MeSH Terms]

12. “laser, ysgg” [MeSH Terms]

13. “laser, argon” [MeSH Terms]

14. “laser, ktp” [MeSH Terms]

15. “laser, q switched” [MeSH Terms]

16. “laser, carbon dioxide” [MeSH Terms]

17. “laser, co2” [MeSH Terms]

18. “laser, diode” [MeSH Terms]

19. “thullium laser”

20. “fluoride laser”

21. “fractional laser”

22. “fractional CO2 laser”

23. “non-ablative fractional laser”

For peer review only

24.“Humans[Mesh]

25. "last 5 years"[PDat]

26. 6 or 7 or 8 or 9 or 10 or 11 or 12 or 13 or 14 or 15 or 16 or 17 or 18 or 19 or 20 or 21 or 22 or 23

26. 5 and 26

Embase:

1. #1, Skin.mp. or exp skin/

2. #2, cutaneous.mp.

3. #3, dermatology.mp. or exp dermatology/

4. #4, skin diseases.mp. or exp skin disease/

5. #5, laser.mp. or exp laser/

6. #6, laser treatment.mp.

7. #7, laser therapy.mp.

8. #8, skin laser therapy.mp.

9. #9, exp argon laser/ or exp frequency doubled neodymium YAG laser/ or exp thulium YAG laser/ or exp dye laser/ or exp gallium aluminum arsenide laser/ or exp neodymium laser/ or exp pulsed dye laser/ or exp carbon dioxide laser/ or exp excimer laser/ or exp YAG laser/ or exp alexandrite laser/ or exp argon fluoride laser/ or exp gas laser/ or exp laser surgery/ or exp erbium YAG laser/

10. #10, nd YAG laser.mp.

11. #11, non-ablative fractional laser.mp.

12. #12, CO2 laser.mp.

13. #13, fractional CO2 laser.mp.

14. #14, carbon dioxide laser.mp. or exp carbon dioxide laser/

15. #15, q switched laser.mp.

16. #16, nd YAG laser.mp.

17. #17, exp symptom assessment/ or exp symptom/ or symptoms.mp.

18. #18, outcome assessment.mp. or exp outcome assessment/

19. #19, treatment outcome.mp. or exp treatment outcome/

For peer review only

20. #20, exp treatment outcome/ or exp outcome assessment/ or outcome.mp.

21. #1 or #2 or #3 or #4

22. #5 or #6 or #7 or #8 or #9 or #10 or #11 or #12 or #13 or #14 or #15 or #16

23. #17 or # 18 or #19 or #20

24. #21 and #22

25. #23 and #24

26. 25 and 2013:2017.(sa_year).

27. 26 and "human" [Subjects]

For peer review only

SUPPLEMENTARY FILE 4

A list of invited patient support groups for the Delphi survey

**Name of Society**
Hidradenitis Patiëntenvereniging (NL)
Nevus Netwerk Nederland (NL)
Nevus Outreach (US)
Nevus Support (AU)
Neurofibromatose Vereniging Nederland (NL)
The Neuro Foundation (UK)
Neurofibromatose Ireland Association (IE)
Vereniging Wijnvlek Sturgeweber syndroom (NL)
Schweizerischen Nuerofibromatose Vereinigung (CH)
Interessengemeinschaf Sturge-Weber-Syndrom (DE)
Sturge Weber Foundation Great Britain (UK)
Sturge-Weber-Foundation (US)
Vitiligo patientenvereniging (NL)
National Vitiligo Foundation (US)

For peer review only
