# Supplementary material for: Generic outcome set for the international registry on Laser trEAtments in Dermatology (LEAD): a protocol for a Delphi study to achieve consensus on what to measure
Source: BMJ Open. 2020 Jun 28;10(6):e038145. doi: 10.1136/bmjopen-2020-038145 (PMC7322331; doi:10.1136/bmjopen-2020-038145)
Supplement: Reviewer comments [file bmjopen-2020-038145.reviewer_comments.pdf]

PEER REVIEW HISTORY

BMJ Open publishes all reviews undertaken for accepted manuscripts. Reviewers are asked to complete a checklist review form (**http://bmjopen.bmj.com/site/about/resources/checklist.pdf**) and are provided with free text boxes to elaborate on their assessment. These free text comments are reproduced below.

ARTICLE DETAILS

**TITLE (PROVISIONAL)**	A generic outcome set for the international registry on Laser TrEAtments in Dermatology (LEAD): a protocol for a Delphi study to achieve consensus on what to measure
**AUTHORS**	Fransen, Frederike; Spuls, Phyllis; Alam, Murad; Badawi, Ashraf; Boixeda, Pablo; Haedersdal, Merete; Hamzavi, Iltefat; Hedelund, Lene; Kelly, Kristen; Kono, Tara; Laubach, Hans Joachim; Manuskiatti, Woraphong; Marini, Leonardo; Nouri, Keyvan; Paasch, Uwe; Passeron, Thierry; Prinsen, C; Verner, Ines; Wolkerstorfer, Albert

This article was not externally reviewed at BMJ Open. Protocols that have been independently assessed prior to submission to BMJ Open are usually fast-tracked to publication on the grounds that further substantial changes will not be possible. This independent assessment will usually be external, independent review for both a substantial grant award from a non-commercial or government funder and ethics approval.
